# Data‐Driven Engineering of Phages with Tunable Capsule Tropism for *Klebsiella pneumoniae*

**DOI:** 10.1002/advs.202309972

**Published:** 2024-06-27

**Authors:** Chao Wang, Shiwei Wang, Shisong Jing, Yuan Zeng, Lili Yang, Yongqi Mu, Zixuan Ding, Yuqin Song, Yanmei Sun, Gang Zhang, Dawei Wei, Ming Li, Yingfei Ma, Haijian Zhou, Linhuan Wu, Jie Feng

**Affiliations:** ^1^ State Key Laboratory of Microbial Resources, Institute of Microbiology, Chinese Academy of Sciences Beijing 100101 China; ^2^ Key Laboratory of Resources Biology and Biotechnology in Western China, Ministry of Education, Provincial Key Laboratory of Biotechnology of Shaanxi Province, the College of Life Sciences, Northwest University Xi'an 710069 China; ^3^ College of Life Science University of Chinese Academy of Sciences Beijing 100049 China; ^4^ Shandong First Medical University & Shandong Academy of Medical Sciences Jinan 250117 China; ^5^ CAS Key Laboratory of Quantitative Engineering Biology, Shenzhen Institute of Synthetic Biology, Shenzhen Institute of Advanced Technology, Chinese Academy of Sciences Shenzhen 518000 China; ^6^ State Key Laboratory for Infectious Diseases Prevention and Control National Institute for Communicable Disease Control and Prevention Chinese Center for Disease Control and Prevention Beijing 102206 China

**Keywords:** antimicrobial resistance, host‐range, phages, receptor‐binding proteins, synthetic biology

## Abstract

*Klebsiella pneumoniae*, a major clinical pathogen known for causing severe infections, is attracting heightened attention due to its escalating antibiotic resistance. Phages are emerging as a promising alternative to antibiotics; however, their specificity to particular hosts often restricts their use. In this study, a collection of 114 phages is obtained and subjected to analysis against 238 clinical *K. pneumoniae* strains, revealing a spectrum of lytic behaviors. A correlation between putative tail protein clusters and lysis patterns leads to the discovery of six receptor‐binding protein (RBP) clusters that determine host capsule tropism. Significantly, RBPs with cross‐capsular lysis capabilities are identified. The newly‐identified RBPs provide a toolbox for customizing phages to target diverse capsular types. Building on the toolbox, the engineered phages with altered RBPs successfully shifted and broadened their host capsule tropism, setting the stage for tunable phage that offer a precise and flexible solution to combat *K. pneumoniae* infections.

## Graphical abstract

The analysis of 114 phages against 238 clinical *Klebsiella pneumoniae* strains produces a phage‐host interaction matrix. This leads to the discovery of six receptor‐binding protein (RBP) clusters targeting specific capsule type and multivalent RBPs with cross‐capsular lysis potential. Utilizing this data, phages are engineered with customized RBPs, effectively shifting or broadening their host range, advancing customizable phage therapy options against *K. pneumoniae*.

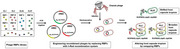

## 1 Introduction

Antimicrobial resistance (AMR) poses a major threat to human health. A recent report estimated that 1.27 million deaths were attributable to AMR worldwide in 2019.^[^ ^1^ ^]^ The AMR crisis is making it imperative to develop alternative treatments, and one of the most promising avenues is phage therapy. Phages are natural predators of bacteria and are unaffected by antibiotic resistance,^[^ ^2^ ^]^ therefore can functional orthogonally on antibiotics to kill bacteria. In phage therapy, the host specificity of phages is a double‐edged sword.^[^ ^3^ ^]^ Phages infect particular bacterial strains, which is beneficial to preserving the natural microbial community and lowering the probability of community‐wide resistance. However, this can also present a significant challenge for phage therapy, as it is crucial to determine the susceptibility of a specific bacterial target to a particular phage prior to treatment. The prevailing method involves combining phages with varied host ranges into a single cocktail to expand the target spectrum. Although this strategy is promising, it remains difficult to target all bacterial strains in a given species. Additionally, assembling multiple phages into a cocktail and optimizing the proportions of phages within a cocktail enhances the complication of therapy. An alternative approach could involve assembling a more uniform set of phages based on common scaffolds and host ranges that are shifted, expanded, or both.^[^ ^4^ ^]^

Receptor‐binding proteins (RBPs) play a central role in host‐range modulation, specifically recognising and binding cell surface receptors to initiate infection.^[^ ^5^ ^]^ The RBPs of some classical phages, such as T3, T4, T7, and λ, have been investigated extensively.^[^ ^5^, ^6^ ^]^ Synthetic biologists implemented design principles from the RBPs of these classical phages and created a tunable host range phage.^[^ ^7^ ^]^ Ando et al. developed a method to alter the host specificity of phages by swapping out RBP or the RBP's C‐terminal region between the *E. coli* phages T3 and T7^[^ ^6^ ^]^ This genetic engineering was accomplished using a yeast‐based recombination platform. The study also showed that exchanging RBP genes between distantly related phages allowed for the genetic modification of an *E. coli* phage to effectively target *Klebsiella* bacteria, and a genetically modified *Klebsiella* phage to effectively target *E. coli* bacteria. Recently, Yehl et al. employed a targeted mutagenesis method inspired by antibody engineering to introduce functional diversity within unstructured loops situated at the RBP‐host cell interface of *E. coli* phage T3. The synthetic “phagebody” libraries generated from this approach (comprising 10^7^ variants) contained individual phages with expanded host ranges and effectively inhibited the development of phage resistance.^[^ ^8^ ^]^ It is obvious that the success of these innovative rational design principles relies on a thorough understanding of the molecular interactions governing specific phage‐host relationships. Therefore, the development of high‐throughput methods for identifying phage RBPs and their conserved structural domains is essential for the rapid engineering of modular phages on a scale suitable for therapeutic applications.^[^ ^7^ ^]^

*Klebsiella pneumoniae* is an important clinical pathogen that can cause severe organ infections.^[^ ^9^ ^]^ The ability to acquire new genetic material enables *K. pneumoniae* to evolve multi‐drug resistance (MDR) and hypervirulence. MDR strains, especially carbapenem‐resistant *K*. *pneumoniae* (CRKP), have become an urgent public health concern, as demonstrated by their inclusion in the priority list of antimicrobial‐resistant pathogens that require new control strategies compiled by the World Health Organization (WHO).^[^ ^10^ ^]^ The development of phage therapy is urgently needed to provide a novel and effective therapeutic option for treating MDR *K. pneumoniae* infection. However, the diversity and complex population structure of *K. pneumoniae* pose challenges to the identification of suitable phages for therapy. This species has 77 capsular (K) serotypes, 11 O serotypes, and hundreds of sequence types (STs), with deep‐branching phylogenetic lineages among *K. pneumoniae* populations and variable AMR profiles.^[^ ^11^ ^]^ Therefore, the identification of *K. pneumoniae* phage RBPs and their conserved structural domains will be crucial in supporting the advancement of phage therapy cocktails and the engineering of phages to broaden their host range. This will ultimately enhance the potential of phages to effectively combat *K. pneumoniae* infection challenges.

In this study, we identified phage RBPs based on large‐scale analysis of bacteria–phage interactions. First, we isolated 114 phages with non‐redundant sequences to construct a phage library. Subsequently, the host range and potency of the phages were evaluated via spot tests against an internal panel of 238 phylogenetically diverse *K. pneumoniae* strains. These lysis modes were classified and found to be associated with specific putative RBPs. Combining these findings with molecular experiments, we identified four clusters of RBPs that determine the tropism for specific host capsule types. Furthermore, we also identified RBPs from generalist phages that are capable of recognizing multiple bacterial capsules. Ultimately, through the application of this uniform set of RBPs, we successfully engineered phages with adjustable host specificity, enabling precise targeting of the *K. pneumoniae* capsule and consequently altering or expanding their host range capabilities.

## 2 Results

### 2.1 Phage Bank Construction

**Figure 1:**
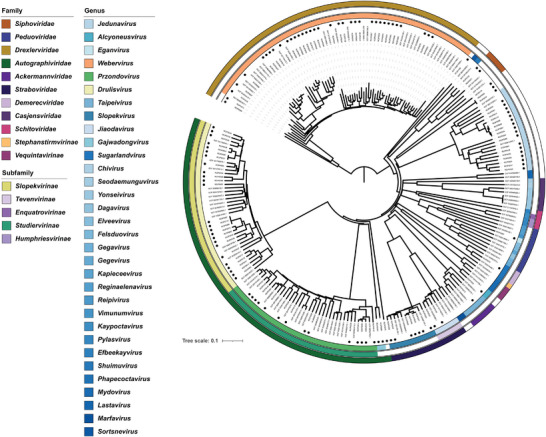
Neighbor‐joining phylogenetic tree constructed from the analysis of 114 phages isolated in this study, alongside 121 *Klebsiella*‐specific phages sourced from the NCBI RefSeq database. The tree is based on the Dice similarity coefficient. Each dot signifies one of the 114 phages examined in our study. The concentric color bands surrounding the tree denote the taxonomic classification of the phages, with the innermost strip representing the genus, the middle strip the subfamily, and the outermost strip the family.

To construct a *K. pneumoniae* phage bank representing a wide host range, we employed a set of 39 *K. pneumoniae* strains containing 15 capsular loci (KLs) and 23 STs as baits for phage isolation, which included most epidemic clones. After intensive isolation efforts from hospital wastewater and domestic sewage, over 200 phages were obtained. Genomic sequencing identified 114 dsDNA *Caudovirales* phages with non‐redundant sequences, with genomic sizes ranging from 27814 to 350 174 bp. Genome annotation using multiple databases showed that these phages harbour 48–634 predicted coding sequences and contain no antibiotic resistance genes or virulence factors. Three of the phages (RCIP0057, RCIP0098, and RCIP0114) encoded integrase genes, indicating that they are temperate phages. To evaluate phage phylogeny, we retrieved 121 *K. pneumoniae* phage genomes from the National Center for Biotechnology Information (NCBI) Refseq database, combining the genomes from our phage bank to construct a protein distance tree (**Figure** **1**). The phages in our bank represented seven families (*Ackermannviridae*, *Autographiviridae*, *Demerecviridae*, *Drexlerviridae*, *Peduoviridae*, *Siphoviridae*, and *Straboviridae*) and ten genera (*Taipeivirus*, *Drulisvirus*, *Przondovirus*, *Sugarlandvirus*, *Webervirus*, *Eganvirus*, *Jiaodavirus*, *Slopekvirus*, *Alcyoneusvirus*, and *Jedunavirus*), which covers most reported *Klebsiella* phage families and genera.^[^ ^12^ ^]^ The phylogenetic classification was supported by morphological observation using transmission electron microscopy (TEM), and the diverse morphologies of myoviruses (*n* = 8), siphoviruses (*n* = 7), and podoviruses (*n* = 4) observed are illustrated in Figure S1 (Supporting Information).

In addition, 61 phages displayed average nucleotide identity (ANI) values <95% with reported genomes, indicating that these phages may represent novel species. To further elucidate the phylogenetic positions of the novel species, genomes of 114 strains were uploaded to Viptree and a protein‐based phylogenetic tree was constructed from the relationships among all phages (Figure S2, Supporting Information). Four of the potentially novel phages (RCIP0034, RCIP0098, RCIP0114, and RCIP0100) were located on three independent branches, indicating that these taxonomically ambiguous phages could represent three novel families, in accordance with the results of vContact clustering (Figure S3, Supporting Information). In summary, the phage bank constructed in this study encompassed most previously described families of *Klebsiella* phages. Furthermore, as numerous potential novel species and even families of *K. pneumoniae* phages were included in this bank, we have substantially increased the phage information landscape for *K. pneumoniae*.

### 2.2 Comprehensive Investigation of Phage‐Host Interactions on a Large Scale

To systematically assess the interactions between *K. pneumoniae* and its phages, we constructed a large host set, which included 238 *K. pneumoniae* strains that were sequenced on the Illumina platform. Based on the core genomes of those 238 strains, a detailed phylogenetic tree was produced (Figure S4A, Supporting Information). Six clades with >90% bootstrap support and dozens of deep‐branching phylogenetic lineages demonstrated the phylogenetic diversity of this strain set. Multi‐locus sequence typing for seven genes (*gap*A, *inf*B, *mdh*, *pgi*, *pho*E, *rpo*B, and *ton*B) and capsular polysaccharide (K) antigen typing for the K locus were performed using Kleborate version v. 2.2.0.^[^ ^11a^ ^]^ These strains belong to 105 STs, of which 94 are known and 11 are unknown (Figure S4B, Supporting Information), and include most pandemic clones, such as ST258, ST11, ST101, ST23, ST65, and ST25, as well as 55 prevalent KLs, including KL1, KL2, KL20, KL47, KL57, and KL64. A search for antibiotic resistance genes (ARGs) in each genome was conducted through ResFinder, resulting in a total of 53 ARGs related to nine classes of antibiotics. The most prevalent ARGs, present in >30% of genomes, confer resistance to β‐lactam, fluoroquinolones, macrolide‐lincosamide‐streptogramin B (MLS_B_), chloramphenicol, sulfonamide, tetracycline, and trimethoprim.

**Figure 2:**
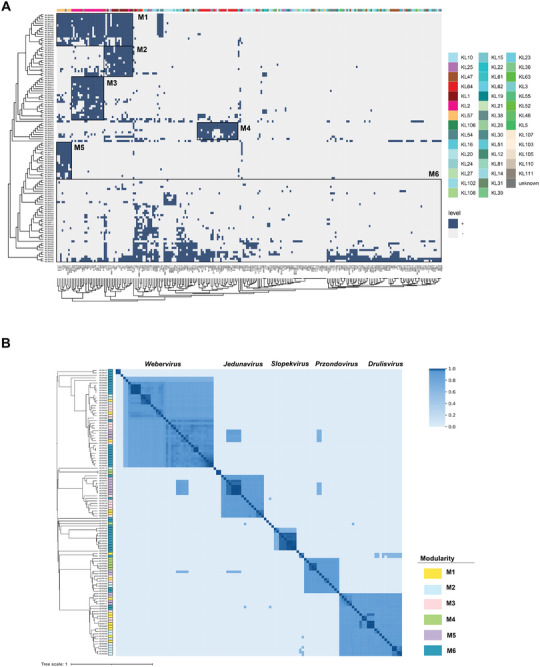
Interactions and genomic relationships of *Klebsiella* phages. A) displays the phage‐bacteria incidence matrix, where 238 *K. pneumoniae* strains were exposed to 114 phages. The matrix is organized with bacterial hosts along the columns and phages along the rows, clustered by default parameters. Blue squares denote successful phage‐host lysis, evidenced by the formation of a single visible plaque. Colored strips above the matrix categorize the *K. pneumoniae* strains by their capsular types (KLs). B) features an ANI heatmap for the 114 phages. Phages are represented along both axes, with the color intensity indicating the ANI value between phage pairs. Accompanying the heatmap is a neighbor‐joining phylogenetic tree of the phage genomes, with color strips marking the lysis module associated with each phage.

The host ranges of each phage in the bank were assayed against these 238 *K. pneumoniae* strains through spot tests. A large phage–host interaction matrix was obtained, which included 3021 lytic phenotypes out of 27132 possible interactions. Overall, 223 of 238 strains (93.70%) were lysed by at least one of 114 phages demonstrating the large capacity of our phage bank. Further examination of the host‐phage bi‐adjacency matrix through hierarchical clustering revealed that lytic interactions segregated into five distinct modules, designated M1 through M5. Notably, these modules showed a strong correlation with the capsular types (KLs) of *K. pneumoniae* strains (**Figure** **2**A). These five typical modules could be divided into two groups according to the coverage of host KLs. One group, exhibiting a broad host range, contained only M1, which harbors 15 phages that can each lyse multiple KLs, mainly KL1, KL2, and KL57. Another group with high specificity for KLs contains the modules M2, M3, M4, and M5. M2 contains 14 phages that mainly lyse KL1, M3 consists of 20 phages lysing KL2, M4 contains eight phages that lyse KL64, and M5 includes 17 phages that lyse KL57. Notably, 40 phages did not cluster corresponding to the KLs of their hosts, and these phages were designed M6.

To assess the infectivity of our phages against specific strains, we randomly selected 353 lytic phenotypes across five distinct modules (M1, M2, M3, M4, and M5) that were initially identified in the spot test. We then applied the double‐layer agar method to validate the infectivity of our phages for a comprehensive evaluation. We observed that 91.22% (322/353) of the interactions recorded in the spot test aligned with the outcomes from the double‐layer agar experiments, as illustrated in Figure S5 (Supporting Information). This high level of consistency highlights the spot test's efficacy in accurately reflecting phage‐bacteria interactions demonstrating its utility for large‐scale evaluations.

### 2.3 Diverse Lytic Modes Exhibited by Phages within the Same Genera

To clarify the correlation between the phylogeny of phages and the lytic module, a whole‐genome phylogenetic tree for the 114 phages in this study was constructed and the ANI for each phage was calculated. Five clusters (each with more than five strains) corresponding to five genera (*Webervirus*, *Jedunavirus*, *Przondovirus*, *Drulisvirus*, and *Slopekvirus*) were observed, and each genus contained strains with close phylogenetic relationships (ANI > 70%) (Figure 2B). Ninety‐seven phages belonging to these five genera were selected for further study. Interestingly, phages within the same genera exhibited differing host ranges with diverse KLs. Specifically, phages of the genera *Webervirus* and *Drulisvirus* displayed the lysis modules of M1, M2, M3, M5, and M6; *Jedunavirus* contained lysis modules M1, M3, M5, and M6; and *Przondovirus* possessed all identified lysis modules. Notably, *Slopekvirus* was an exception, with seven of its phage strains demonstrating no tropism related to host serotype. Collectively, these results indicate that phage lysis patterns show no apparent correlation with phage phylogeny.

### 2.4 Correlation between Putative Tail Protein Clusters and Lysis Modules Identified

**Figure 3:**
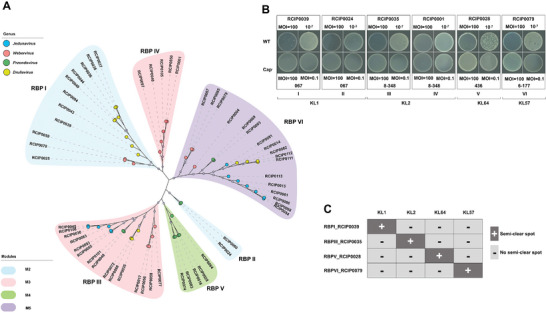
Phylogenetic analysis of receptor‐binding proteins and functional characterization of *Klebsiella* phages. A) Phylogenetic analysis of RBPs. This panel features a maximum‐likelihood phylogenetic tree of the putative RBPs from phages in lysis modules M2, M3, M4, and M5. Bootstrap support values exceeding 90% are demonstrated at the corresponding nodes. B) Double‐layer plaque assay of the infectivity of *Klebsiella* Phages from M2 to M5 against wild type (WT) and acapsular mutant (Cap^−^). Three independent experiments were conducted, and a representative result is shown. C) Activity of purified RBPs on *K. pneumoniae* lawns. Spot tests were performed using purified RBPs on lawns of *K. pneumoniae*. The ORF of RBPI from phage RCIP0039, RBPIII from RCIP0035, RBPV from RCIP0028, and RBPVI from RCIP0079 were selected for expression and purification to assess their functional activity.

Phages recognise their hosts through RBPs, which form fibres or spikes at the distal phage tail. RBPs bind to specific surface receptors of bacteria that may be outer membrane proteins, lipopolysaccharides, or components of bacterial capsules, pili or flagella.^[^ ^5^ ^]^ To resolve the factors that determine the phage lysis modules in this study, we conducted a comparative genomic analysis of phages M2–M5, which belong to the same genus, to identify the RBPs responsible for determining their tropism. We aligned the contiguous cluster encoding morphogenesis proteins from the genera *Webervirus*, *Jedunavirus*, *Przondovirus*, and *Drulisvirus* (Figure S6, Supporting Information). Interestingly, the genes encoding morphogenesis proteins shared >70% nucleotide identity within a given genus, except for one gene, the predicted phage tail protein gene. Within a genus, putative phage tail proteins of phages in the same lysis module shared high identity. A phylogenetic tree was constructed based on 55 putative phage tail protein sequences identified in this study. The proteins formed six monophyletic clades, which were designated Clusters I, II, III, IV, V, and VI (**Figure** **3**A). Cluster I of the genera *Drulisvirus* and *Webervirus* and Cluster II of *Przondovirus* correspond to the M2 lysis module, which specifically lysed KL1 strains. Cluster III, containing the genera *Drulisvirus*, *Webervirus* and *Jedunavirus*, and Cluster IV of *Przondovirus* correspond to lysis module M3, targeting KL2 strains. Cluster V, containing *Przondovirus*, represent module M4, lysing KL64 strains. Finally, Cluster VI, with *Drulisvirus*, *Przondovirus*, *Webervirus*, and *Jedunavirus* correspond to M5, targeting KL57 strains. The strong correlation between the putative phage tail protein cluster and the lysis module suggests that these phage tail proteins are RBPs responsible for host recognition. Notably, other putative phage tail proteins that were found in the conserved region for a given genus showed no correlation with the lysis module.

### 2.5 Identification of RBPs Determining the Host Capsule Tropism of *Klebsiella* Phages

To address our hypothesis that the putative phage tail proteins function as RBPs that bind the capsule polysaccharide (CPS) of *K. pneumoniae*, we compared the binding of wild‐type strains and acapsular mutants. First, we generated CPS‐deficient mutants of KL1, KL2, KL57, and KL64 through serial passage on Luria–Bertani (LB) plates, respectively. Selected representative phages with putative phage tail proteins from six clusters were used to evaluate infectivity against wild‐type and acapsular mutant strains of *K. pneumoniae* using the double‐layer agar method at MOIs of 100 and 0.1. Phage RCIP0039, with putative phage tail proteins in Cluster I, and phage RCIP0024 from Cluster II formed visible plaques on lawns of KL1 wild‐type strains but not on those of the isogenic acapsular mutant (Figure 3B). Similarly, phages RCIP0035 from Cluster III and RCIP0001 from Cluster IV formed visible plaques on wild‐type KL2 strains but not on the acapsular mutant, and phages RCIP0028 from Cluster V and RCIP0079 from Cluster VI formed visible plaques on wild‐type KL64 and KL57 strains but not the acapsular mutant. These results indicate that some component of the bacterial capsule is the binding receptor of these phages.

To characterise the polysaccharide‐depolymerising function of RBPs in different clusters, candidate genes from Clusters I, III, V, and VI were expressed and purified. The molecular weights of RBPI_RCIP0039, RBPIII_RCIP0035, RBPV_RCIP0028, and RBPVI_RCIP0079 were 69.9, 60.8, 110, and 82.3 kDa (Figure S7, Supporting Information), respectively, and the activities of these recombinant proteins were examined using spot tests on lawns of strains with the KL1, KL2, KL64, and KL57 loci. RBPI_RCIP0039 generated a semi‐clear spot on a lawn of the KL1 strain 8–105 but not on those of strains with other KLs. Similarly, RBPIII_RCIP0035, RBPV_RCIP0028, and RBPVI_RCIP0079 showed semi‐clear spots on the corresponding KL strains, and no spots on lawns with other KLs. Taken together, these results indicated that these RBPs exhibit specificity in polysaccharide depolymerisation (Figure 3C; Figure S8A, Supporting Information).

### 2.6 Discovery of Multivalent RBPs with Cross‐Capsular Lysis Potential

**Figure 4:**
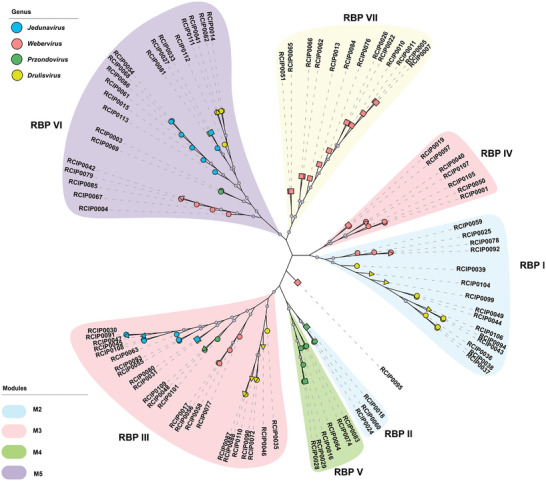
Maximum‐Likelihood phylogenetic tree of putative depolymerase proteins from phages across lysis modules M1–M6. The topology of the tree is annotated with distinct symbols to represent each lysis module: circles denote modules M2–M5, triangles indicate module M1, and squares correspond to module M6. Nodes with bootstrap support values exceeding 90% are demonstrated.

The majority of phages in the genera *Webervirus*, *Jedunavirus*, *Przondovirus*, and *Drulisvirus* in our library lysed specific KL strains. However, there are a few phages were identified in the four genera, belonging to the M1 and M6 lysis modules and exhibiting multi‐capsule tropism. Thirty‐five potential RBPs were identified. A phylogenetic tree was constructed based on the protein sequences of the 55 RBPs described above and these 35 potential RBPs, and seven distinct clusters were observed (**Figure** **4**). Among them, one new cluster was observed and designated Cluster VII. This cluster consisted of 13 potential RBPs from *Webervirus* phages with the M6 lysis module, in addition to the six clusters ascribed to the RBPs noted above. Furthermore, 14 potential RBPs from M1 phages that could lyse cells of KL1, KL2, and KL57 were clustered with specific RBPs in Clusters I, III, IV, and VI. Specifically, potential RBPs from M1 phages shared high amino acid identities (from 90% to 99.47%) with RBPs from specialist phages in the same genus. Similarly, seven potential RBPs with module M6 clustered with specific RBPs in Clusters II, III, IV, V, and VI and shared high amino acid identities (from 97.35% to 99.31%).

Two putative RBPs from phages, associated with the M1 lysis module were expressed. The RBP from phage RCIP0104 falls into Cluster I, which comprises RBPs that specifically target the KL1 capsule type, whereas the RBP from RCIP0046 phage is categorized within Cluster III, which contains RBPs with specificity toward the KL2 capsule type. Assays conducted with the two RBPs demonstrated semi‐clear plaques on bacterial lawns of strains possessing KL1 and KL2 capsules (Figure S8B, Supporting Information). In contrast, no plaques were observed when these RBPs were applied to lawns of the KL57 strains, indicating the RBPs' specificity for the KL1 and KL2 capsule types and their inability to degrade the KL57 capsule polysaccharides. Sequence analysis of RBPs from a broad‐host‐range (generalist) phage and their counterparts from a narrow‐host‐range (specialist) phage revealed high amino acid sequence identity, with RBP‐RCIP0104 (generalist) and RBP‐RCIP0039 (specialist) sharing 96.93% identity, despite 19 amino acid substitutions. Similarly, RBP‐RCIP0046 (generalist) and RBP‐RCIP0035 (specialist) shared 97.23% identity with 16 amino acid differences. These sequence variations may underpin the observed specificity and functional differences between the generalist and specialist RBPs.

### 2.7 Engineered Phages with Shifted Host Capsule Tropism

**Figure 5:**
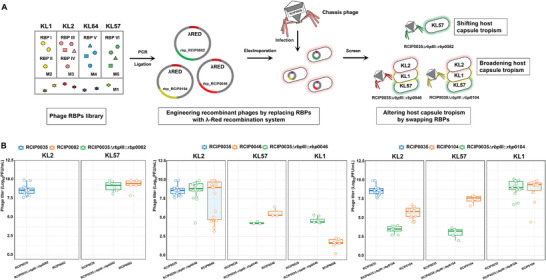
Host capsule tropism alteration by swapping RBPs. A) Schematic showing the strategy to engineer phages; B) Lytic activity assays comparing recombinant phages with their RBP donor phages. The initial titer of phages were normalized to 8 × 10^9^ [PFU]/mL.

The newly identified RBPs provide a toolbox for customizing phages to target diverse capsular types. We hypothesized that swapping the RBP could result in phages with altered host capsule tropism (**Figure** **5**A). To test this hypothesis, we selected phage RCIP0035 from our library. The genome of RCIP0035 is 43 252 bp in length, with a GC content of 54.24%, and it lacks genes encoding tRNA, antibiotic resistance, toxins, virulence factors, or lysogeny‐promoting gene clusters. Genome analysis demonstrated that phage RCIP0035 belongs to the genus *Drulisvirus* within the *Slopekvirinae* subfamily of the *Autographiviridae* family.

Afterward, we successfully engineered a recombinant phage by replacing the RBPIII‐RCIP0035 gene of phage RCIP0035, originally tailored for KL2 capsule type strains, with the RBPVI‐RCIP0082 gene derived from phage RCIP0082, which has specificity for KL57 capsule type strains. This genetic manipulation led to the creation of the hybrid phage designated as RCIP0035∆*rbp*III::*rbp*0082. This novel phage variant demonstrated a shift in host specificity; it lost the capacity to lyse its initial hosts with the KL2 capsule but acquired a new lytic activity against strains harboring the KL57 capsule (Figure 5B).

### 2.8 Engineered Phages with Broaden Host Capsule Tropism

Subsequently, we investigated whether swapping multivalent RBPs with cross‐capsular lysis potential could yield phages with an expanded host capsule tropism. We replaced the RBPIII‐RCIP0035 gene of phage RCIP0035, which is selective for strains with the KL2 capsule type, with RBP genes from phages RCIP0046 and RCIP0104. These donor phages have a broad host range, capable of lysing strains with KL1, KL2, and KL57 capsule types, and RBPIII‐RCIP0046 and RBPI‐RCIP0104 demonstrated capacity to depolymerise KL1 and KL2 capsule types polysaccharides. Notably, both RBPIII‐RCIP0046 and RBPIII‐RCIP0035 belong to cluster III sharing 97.23% sequence identity, whereas RBPI‐RCIP0104 belong to cluster I and displays <30% identity with that of RBPIII‐RCIP0035. The resulting recombinant phages, designated RCIP0035∆*rbp*III::*rbp*0046 and RCIP0035∆*rbp*III::*rbp*0104.

RCIP0035∆*rbp*III::*rbp*0046 and RCIP0035∆*rbp*III::*rbp*0104 were subjected to lytic efficacy tests against a diverse panel of 41 strains, encompassing 14 KL1 strains, 18 KL2 strains, and 9 KL57 strains. The recombinant phage RCIP0035∆*rbp*III::*rbp*0046 not only retained its lytic efficacy against KL2 capsule strains but also expanded its lytic range to include KL1 and KL57 capsule strains, as depicted in Figure 5B. Similarly, RCIP0035∆*rbp*III::*rbp*0104 exhibited a broadened host range, maintaining its activity against KL2 capsule strains and extending its lytic capacity to include both KL1 and KL57 capsule types. Interestingly, the lysis spectrum of the recombinant phages did not completely mirror that of their donor phages. For instance, RCIP0035∆*rbp*III::*rbp*0046 was able to lyse an additional three strains of the KL2 type compared to its donor phage RCIP0046 (Figure 5B). This suggests that the hybrid phages not only acquired the broad host range of their donors but also potentially developed unique lytic properties, underscoring the intricate nature of phage‐host interactions and the potential for engineered phages to exhibit novel and enhanced capabilities in bacterial lysis.

## 3 Discussion

To date, a variety of strategies have been employed to modulate the host ranges of various phages. However, the systematic, efficient, and high‐throughput engineering of phages with desired host ranges has yet to be achieved. Our study represents a significant step forward in this endeavor. In this study, we performed an in‐depth analysis of the interactions between phages and bacteria. Based on the data obtained from these interactions, we mined the host range determinants (RBPs) from the sequence database. Subsequently, we exchanged specific portions of well‐characterized phage scaffolds with the identified RBPs to engineer novel phages with desired host range specificities.

Deciphering the molecular mechanisms underlying phage‐bacteria interactions is fundamental to the engineering of phages with tailored host range specificities. The primary receptor targeted by RBPs of many *Klebsiella* phages is CPS, which is a crucial virulence factor, enabling bacteria to avoid phagocytosis or complement‐mediated killing.^[^ ^13^ ^]^ Differences in sugar composition, namely the specific ratios of various sugar components, as well as variations in locus organisation, are used to distinguish at least 77 capsular serotypes (K antigens) and 134 KLs among *Klebsiella* species.^[^ ^14^ ^]^ A recent study employed 46 phages to challenge 138 representative clinical isolates of *K. pneumonia*, and that found depolymerase sequence types could associate infection specificity with capsular types.^[^ ^12^ ^]^ However, the limited matches of lytic phenotypes between phage and host (124 out of 6348 phage–bacteria pairs) provides insufficient information for classifying these RBPs. In our study, we analyzed an extensive phage‐host interaction matrix, yielding data on 3021 lytic phenotypes from a pool of 27132 potential interactions. By integrating this interaction data with phage genomic sequences, we were able to identify putative RBPs that drive capsule‐specific tropism in *Klebsiella* phages. The subsequent molecular experiments we identified four clusters of specific RBPs that determine the host capsule tropism of *Klebsiella* phages. The newly identified RBPs supply a toolbox for customizing phages to target specific capsular types of *K. pneumoniae*.

The broad host range of *K. pneumoniae* phages has been documented, with many exhibiting multiple RBPs.^[^ ^12^, ^15^ ^]^ It has been observed in several studies that a single RBP gene can recognize multiple serotypes,^[^ ^16^ ^]^ exemplified by phage KpV71, which can concurrently recognize serotypes KL1 and KL62. However, the relationship between RBPs and capsule types has not been thoroughly explored due to the lack of comprehensive analysis of phage‐bacteria interactions based on a diverse array of test strains. By utilizing these test strains, we can identify RBPs and characterize their host range across multiple capsule types. Interestingly, some RBPs from generalist phages show high sequence identity with those from specialist phages, indicating that variations in phage host range could be attributed to minor point mutations. The RBPs identified in this study provide a valuable toolkit for engineering phages to target a wide range of capsular types in *K. pneumoniae*.

Phages' high specificity allows them to target specific bacterial strains, but this means one phage type may not work against all strains in a species. Treating diverse bacterial infections often requires using phage cocktails. Regulatory approval for these cocktails is challenging due to varied phages structures, life cycles, and genomes.^[^ ^17^ ^]^ Traditional methods requiring the screening of phages from a library for each bacterial strain result in a collection of novel phages with poorly characterized features. An alternative strategy is to use a single well‐characterized phage as a foundational platform and engineer it by substituting RBPs.^[^ ^4^ ^]^ Armed with acquired knowledge of RBPs, we manipulate *Klebsiella* phage more efficiently to change their hosts or even expand their host range with RBPs. We successfully engineered phages to alter their host capsule tropism, shifting specificity from KL2 to KL57. Additionally, we expanded the host capsule range of these phages to recognize and infect hosts with KL1, KL2, and KL57 capsules. That is different from alteration of phage host specificity which performed on classical phage, such as T2, T4 and T7, by exchanging tail fiber genes through the process of homologous recombination among phages that share a close genetic relationship, particularly within the T2, T4, and T7 lineages.^[^ ^18^ ^]^ The engineered phages demonstrated the ability to lyse clinical strains of *K. pneumoniae*, rather than merely type strains, showcasing their practical applicability in real‐world scenarios. Furthermore, we employed a single phage as a chassis and altered its capsule tropism solely by replacing characterized RBPs. By applying this strategy, we can create a more uniform set of phages based on common scaffolds, which simplifies the therapeutic approach and enhances the safety of the treatment. The application of engineered broad‐spectrum phages must be undertaken with awareness of the need to protect the host's microbiome integrity while also eradicating pathogen.

Our study provided a novel strategy that allows the creation of a synthetic phage library, where each member has a clearly defined host range, and the properties of the base phage are thoroughly understood. Such an approach streamlines the development of synthetic phage therapies, potentially simplifying the regulatory process by providing a standardized platform with customizable targeting capabilities.

## 4 Experimental Section

### Bacterial Strains and Growth Conditions

*K. pneumoniae* and *Escherichia coli* strains were grown at 37 °C in LB medium (10 g L^−1^ tryptone, 5 g L^−1^ yeast extract, and 10 g L^−1^ NaCl). As needed, the medium was supplemented with kanamycin (50 µg mL^−1^).

### Phage Isolation

Hospital wastewater and domestic sewage samples were collected from four cities in China: Beijing, Nanjing, Xi'an, and Cangzhou. A set of *K. pneumoniae* strains, including 39 strains containing 15 KLs and 23 STs, was used as hosts for phage isolation. Phages were isolated using the double‐layer agar method as previously described.^[^ ^19^ ^]^ This procedure was iterated at least three times to purify each phage. High‐titer phage stocks were produced by soaking multiple plaque on soft agar plates. Briefly, phages were incubated on plates overnight and 5 mL of SM buffer was added to resuspend the phages. After incubating at room temperature for a minimum of 3 h, the suspension was collected and treated with chloroform to lyse bacteria. Subsequently, the solution was filtered through a 0.22 µm filter. The resulting phage titer was quantified using the double‐layer agar method and stored at 4 °C.

### Phage Imaging via Transmission Electron Microscopy

TEM was used to observe phage morphology after isolation.^[^ ^20^ ^]^ Five millilitre of phage particles (≥10^9^ plaque‐forming units [PFU]/mL) were placed on formvar and carbon‐coated copper electron microscopy grids (200 mesh), allowed to adsorb for 20 min, and negatively stained with 1% (w/v) uranvl acetate for 1 min. After the excess stain was removed with filter paper, the grids were air‐dried prior to examination. TEM was conducted at the Institute of Microbiology, Chinese Academic Sciences (Beijing, China) on HT7800 (Hitachi, Japan) and JEM‐1400 (JEDL, Japan) electron microscope at 80 kV. Images were recorded with an AMT‐BioSprint16 and Gatan 832 digital camera. Phage morphology and taxonomic assignment were confirmed following the guidelines of the International Committee on Taxonomy of Viruses (http://www.ictvonline.org/).

### Phage Genome DNA Extraction, Sequencing, and *de novo* Contig Assembly

After phage enrichment in liquid culture using corresponding hosts, the culture was collected and treated with chloroform to lyse bacteria. Subsequently, the solution was filtered through a 0.22 µm filter, and genomic DNA was extracted using Lambda phage Genomic DNA Kit (ZOMANBIO, Beijing, China). The genomic DNA was sequenced at Beijing Novogene Technology Co., Ltd. (Beijing, China). Samples were sequenced on the Illumina HiSeq 2000 platform. After quality filtering, the clean reads were subsampled to a data size of 20 Mbp using the seqtk tool (https://github.com/lh3/seqtk). The reads were then mapped to the genomes of reference bacteria obtained from the gcType database (as of June 2022)^[^ ^21^ ^]^ of the National Microbiology Data Center, using bowtie2^[^ ^22^ ^]^ to remove non‐viral bacterial sequences. The remaining reads were assembled using SPAdes (v3.15.5)^[^ ^23^ ^]^ with the parameters –metaviral and –only‐assembler. Based on the length of the assembled sequences and the read coverage, a unique high‐quality phage genome sequence was attempted to selected. The completeness of the assembled sequences was evaluated using CheckV.^[^ ^24^ ^]^ In cases in which the read coverage was particularly low or the CheckV evaluation indicated a high probability of incompleteness, the subsampling data size was adjusted to 40 Mbp or reassembled reads using SPAdes with the –meta parameter. All assembled contigs were evaluated using CheckV and showed high confidence of being high‐quality or complete phage genomes (completeness > 90%). Error correction for each genome was performed using the Pilon tool.^[^ ^25^ ^]^

### Phage Genome Annotation and Similarity Analysis

The open reading frames (ORFs) of phages were predicted and annotated using Prokka software (v.1.14.6)^[^ ^26^ ^]^ with the parameters –kingdom viruses and –metagenome. The protein sequences thus obtained were further annotated using the following methods: submitting sequences to the NCBI Batch CD‐search web‐server with the Pfam and TIGR databases specified,^[^ ^27^ ^]^ submitting the sequences to the KofamKOALA web‐server based on the Kyoto Encyclopedia of Genes and Genomes database,^[^ ^28^ ^]^ and performing a BLASTp search between phage genomes and sequences downloaded from VOGDB (https://vogdb.org/) with thresholds of identity >40% and coverage >40%.

Inter‐genomic similarities were identified using BLASTn pairwise comparisons. Virus assignment into genera (≥70% similarity) and species (≥95% similarity) ranks followed the International Committee on Taxonomy of Viruses (ICTV) genome identity thresholds. ANI was calculated using the Python package pyani (v.0.2.11) (http://huttonics.github.io/pyani). The ANI results were then visualized based on the order of the phylogenetic tree. Clusters of phage genomes were generated using vConTACT2 (v.0.11.3)^[^ ^29^ ^]^ with the reference database ProkaryoticViralRefSeq211‐Merged. Specifically, edges in the network file were filtered out when the nodes on both ends of the edge belonged to none of the 114 phages in this study and did not overlap or belong to the same viral cluster as these phages. Finally, the results were visualized using Cytoscape.^[^ ^30^ ^]^

### Phylogenetic Analysis of Phages

To clarify the phylogenetic level of the novel species, a phylogenetic tree based on genome‐wide pairwise distances was generated using the Viral proteomic tree (ViPTree) server.^[^ ^31^ ^]^ A subset of 1468 closely related reference genomes in ViPTree server, together with genomes of 114 phages newly sequenced in this study was used to generate the phylogenetic tree. In addition, 121 reference genomes of phages infecting *K. pneumonia*e were downloaded from the NCBI RefSeq database.^[^ ^32^ ^]^ Basing on the protein sequences of these 121 *Klebsiella* phages and our 114 phages, an all‐versus‐all comparison was performed through Diamond^[^ ^33^ ^]^ (v.2.0.7) with the more‐sensitive mode. Then, a Dice distance matrix was calculated using the formula reported previously.^[^ ^34^ ^]^ That matrix was used to generate a neighbour‐joining tree using the R package ape (v.5.5).^[^ ^35^ ^]^

### Molecular Typing and Phylogenetic Analysis of *K. pneumoniae* Genomes

Genomic features, including KL types, multi‐locus sequence typing (MLST), virulence factors, and antibiotic resistance loci of the assembled *K. pneumoniae* genomes were predicted using Kleborate (v.2.2.0).^[^ ^11a^ ^]^ Then, the general feature format (gff) files of 238 *K. pneumoniae* genomes produced by Prokka (v1.14.6)^[^ ^26^ ^]^ were used as the inputs to Roary (v3.13.0)^[^ ^36^ ^]^ to create a core gene alignment. A maximum‐likelihood tree was constructed based on that alignment using RAxML (v8.2.12)^[^ ^37^ ^]^ with the GTRGAMMA method.

### Host‐Range Determination

The isolated phages were tested against *K. pneumoniae* strains (n = 238). Phage lytic activity was determined through a spotting technique, involving co‐incubation of phages and bacteria on LB agar for 18 h. Each phage lysate was spotted (10^9^ [PFU]/mL, 3 µL) onto the selected hosts. Phage activity against each strain was determined to be either lysed, with clear plaques, or non‐lysed, with no visible plaques. A phage–bacteria incidence matrix was plotted using the heatmap.2 function of the ggplots R package.

To validate the spotting assays results, 353 lytic phenotypes were randomly selected from five distinct modules (M1, M2, M3, M4, and M5) identified in the spot test then validated the infectivity of our phages through the double‐layer agar method.

### Analyzing the Role of Putative RBPs in Determining Capsule Tropism in *K. pneumoniae*

The phages belonging to M2–M5 were selected, for analysis. The contiguous clusters encoding morphogenesis proteins are aligned from each of the genera, including *Webervirus, Jedunavirus, Przondovirus*, and *Drulisvirus*, to identify the putative RBPs responsible for determining their tropism. The genes encoding morphogenesis proteins were compared using BLAST and visualised with Easyfig.^[^ ^38^ ^]^ The RBPs were aligned using MAFFT (v.4.787)^[^ ^39^ ^]^ and trimmed using trimAl (v1.4.rev15).^[^ ^40^ ^]^ The maximum‐likelihood tree was constructed using IQtree (v.2.1.4‐beta)^[^ ^41^ ^]^ based on the trimmed alignment of the putative RBPs.

### In Vitro Expression of Phage RBPs and Activity Assay

To evaluate the functions of putative RBPs, RBPs from different lysis module phages targeting various KLs were expressed in vitro. The gene of RBPs from six phages were cloned and inserted into the vector pET28a via the *Nde*I and *Xho*I restriction sites. The primers used to construct the expression vectors are listed in Table S1 (Supporting Information). The recombinant plasmids were transformed into *E. coli* BL21(DE3). A fresh overnight culture was used to inoculate 500 ml LB media with 50 µg mL^−1^ kanamycin in a 1/100 inoculum size. Culture was incubated at 37 °C up to the log phase (OD_600nm_ ≈ 0.5), followed by continued cultivation by addition of 0.5 mm isopropyl‐β‐d‐thiogalactopyranoside for an additional 18 h at 16 °C. The His‐tagged recombinant protein was purified using nickel beads (Beyotime, Shanghai, China) according to the manufacturer's instruction manual. After the expression and purification of RBPs were performed as previously described,^[^ ^15^, ^42^ ^]^ the functions of RBPs were measured by dotting plates with various bacterial indicators. Specifically, 200 µL of fresh bacterial culture was mixed with 8 mL of semi‐solid LB and placed on the surface of an LB agar plate. Aliquots of various concentrations of purified recombinant RBPs were spotted onto the plate after the top agar layer had solidified. After incubation at 37 °C for 6–8 h, semi‐clear spots were observed.

### Construction of Phages with Shifted and Broadened Host Capsule Tropism by Replacement of RBP

Engineered phages were developed through the replacement of the RBPs utilizing a method adapted from a previously established approach.^[^ ^15^ ^]^ Specifically, the L‐arabinose‐inducible λ‐Red recombination system^[^ ^43^ ^]^ was employed and integrated it into the pCOLADuet‐1 vector (Novagen, Darmstadt, Germany), which was equipped with a kanamycin resistance marker. This vector was capable of replicating in both *E. coli* and *K. pneumoniae* strains, resulting in plasmid pCOLADRed (Table S3 and Figure S9, Supporting Information). To construct the fusion fragments, the Gibson assembly technique (New England Biolabs, Ipswich, MA, USA) was applied. The chassis phage used in this process was phage RCIP0035. This involved the amplification of both the upstream and downstream regions (≈500 bp each) surrounding the RBPIII‐RCIP0035 gene, along with the RBP genes intended to replace the RBPIII‐RCIP0035 gene (Table S2, Supporting Information). Subsequently, these fragments were introduced into pCOLADRed, generating plasmids pCOLADRedRE0082, pCOLADRE0046, and pCOLADRE0104 (Table S3, Supporting Information). These plasmids were individually transformed into the Kp7‐50 host strain and then screened on LB agar plates containing 50 µg mL^−1^ kanamycin.

Strains of Kp7‐50 harboring the recombinant plasmids were induced using 1 mm arabinose to express the recombinase system. Cultures of these bacteria in the log phase (OD_600_nm ≈ 0.5) were co‐incubated with various concentrations of phage RCIP0035 for 30 min at 37 °C. The mixtures were then spread onto double‐layer agar plates for incubation overnight. From plates showing a moderate number of plaques (≈20), single plaques were selected for PCR analysis to confirm the presence of recombinant phages carrying the introduced RBP genes. To mitigate the risk of contamination with wild‐type phage, a subsequent round of infection was carried out using plaques that tested positive in the initial PCR screening. Following this, single plaques were once again subjected to PCR analysis to verify successful recombination. The sequencing data confirming these results are depicted in Figure S10 (Supporting Information).

## Conflict of Interest

The authors declare no conflict of interest.

## Supporting information

Supporting Information

## Data Availability

The data that support the findings of this study are openly available in National Microbiology Data Center at https://nmdc.cn/, reference number 60139380.
