# Supplementary material for: Data‐Driven Engineering of Phages with Tunable Capsule Tropism for Klebsiella pneumoniae
Source: Adv Sci (Weinh). 2024 Jun 27;11(33):2309972. doi: 10.1002/advs.202309972 (PMC11434222; doi:10.1002/advs.202309972)
Supplement: Supplementary file 1 — Supporting Information [file ADVS-11-2309972-s001.pdf]

ADVANCED SCIENCE
Open Access

# Supporting Information

for *Adv. Sci.*, DOI 10.1002/advs.202309972

Data-Driven Engineering of Phages with Tunable Capsule Tropism for *Klebsiella pneumoniae*

*Chao Wang, Shiwei Wang, Shisong Jing, Yuan Zeng, Lili Yang, Yongqi Mu, Zixuan Ding, Yuqin Song, Yanmei Sun, Gang Zhang, Dawei Wei, Ming Li, Yingfei Ma, Haijian Zhou*, Linhuan Wu* and Jie Feng**

# Supporting Information

## Data-Driven Engineering of Phages with Tunable Capsule Tropism for *Klebsiella pneumoniae*

Chao Wang[1,7], Shiwei Wang[3,7], Shisong Jing[1,4,7], Yuan Zeng[1], Lili Yang[1,5], Yongqi Mu[1,4], Zixuan Ding[1,5], Yuqin Song[1], Yanmei Sun[3], Gang Zhang[1], Dawei Wei[1], Ming Li[1], Yingfei Ma[6], Haijian Zhou[2,8], Linhuan Wu[1,8], Jie Feng[1,8]

Fig S1-S10

Table S1-S3

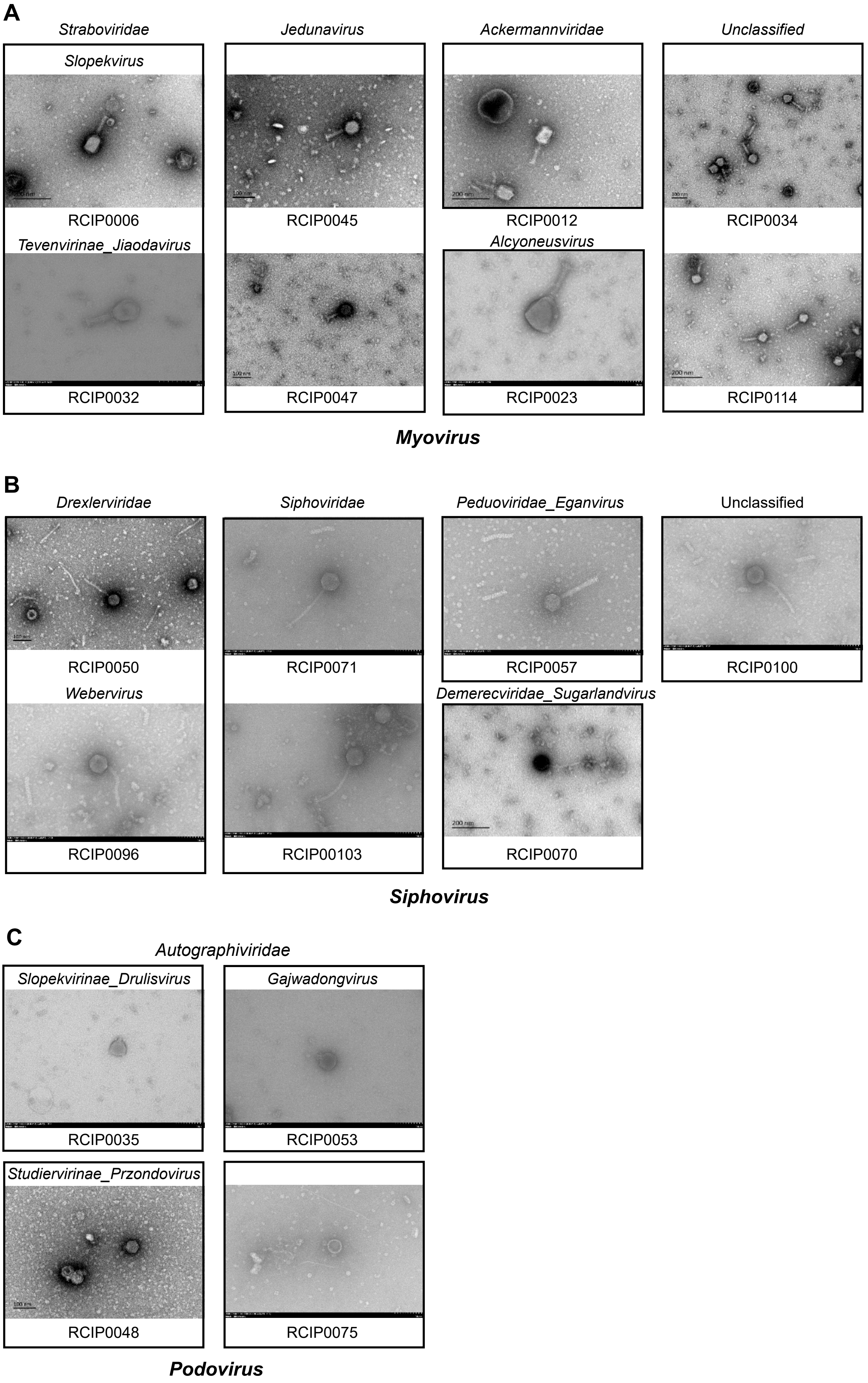


Figure S1. Electron micrographs of representative *Klebsiella* phages from each family.

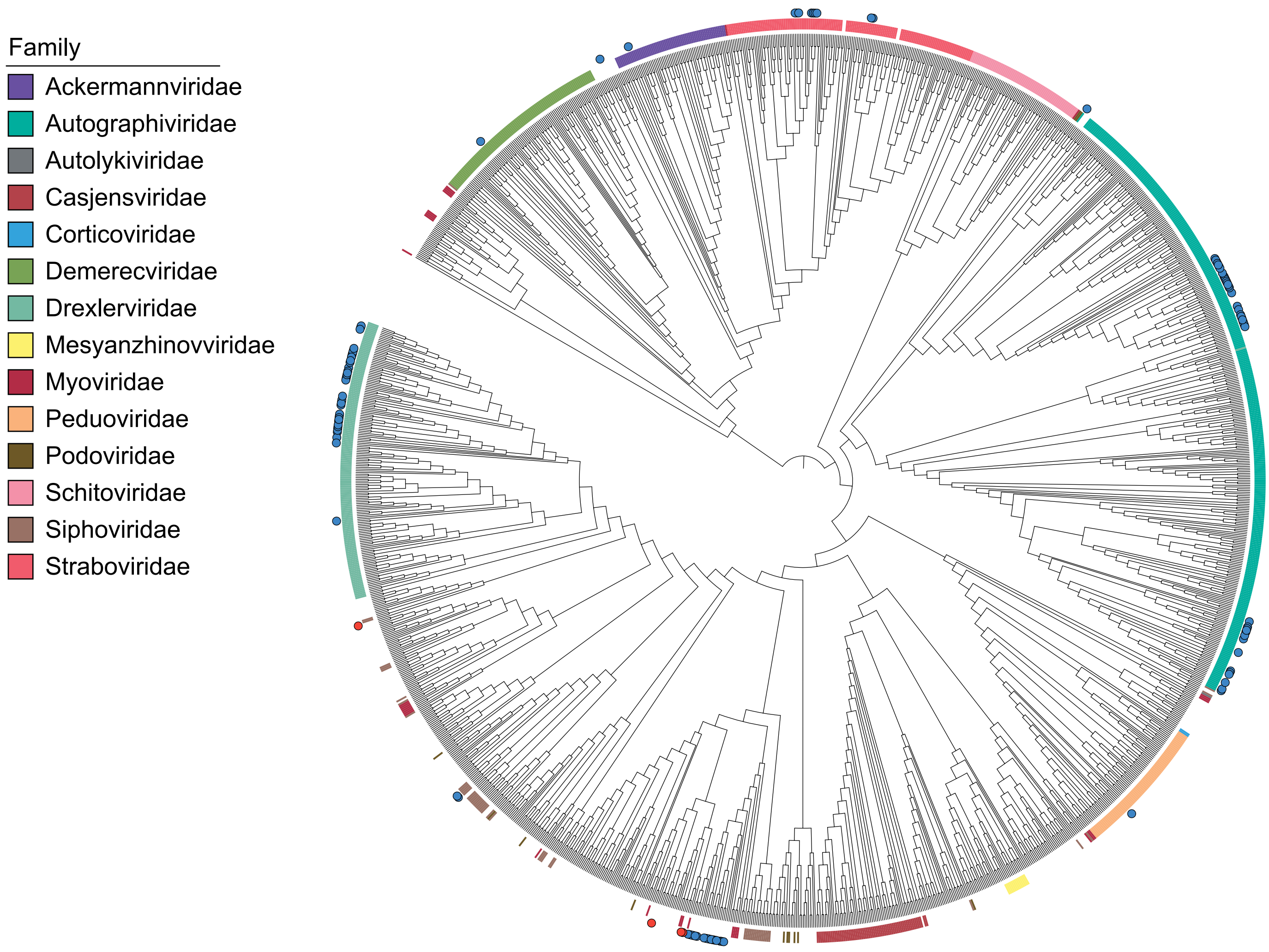


Figure S2. Phylogenetic tree constructed using ViPTree for 114 phages in this study and 1,468 refer-ence phages. The color strips represent the phage family. Blue dot represents the 110 phages of this study. Red dot represents the 4 potentially novel familly phages of this study.

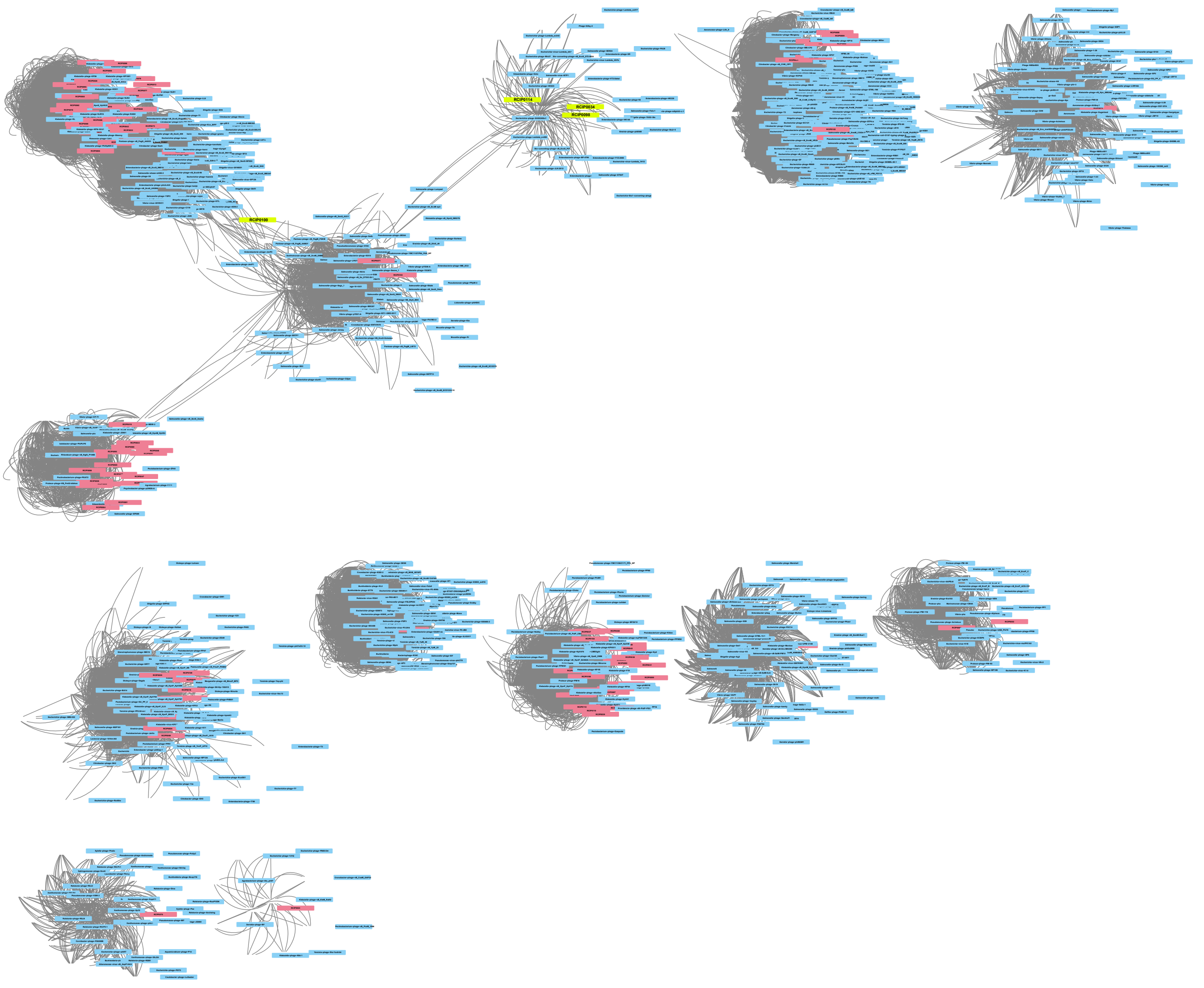

Figure S3. Clustering obtained using vContact2 for 114 phages and references from RefSeq. Pink labels represent the 110 phages of this study, yellow labels represent 4 potentially novel familly phages of this study, and blue labels represent the phages from RefSeq. Some references that had weak associations with the 114 phages in this study were removed from the figure. The length of each edge reflects the strength of the connectivity between two phages.

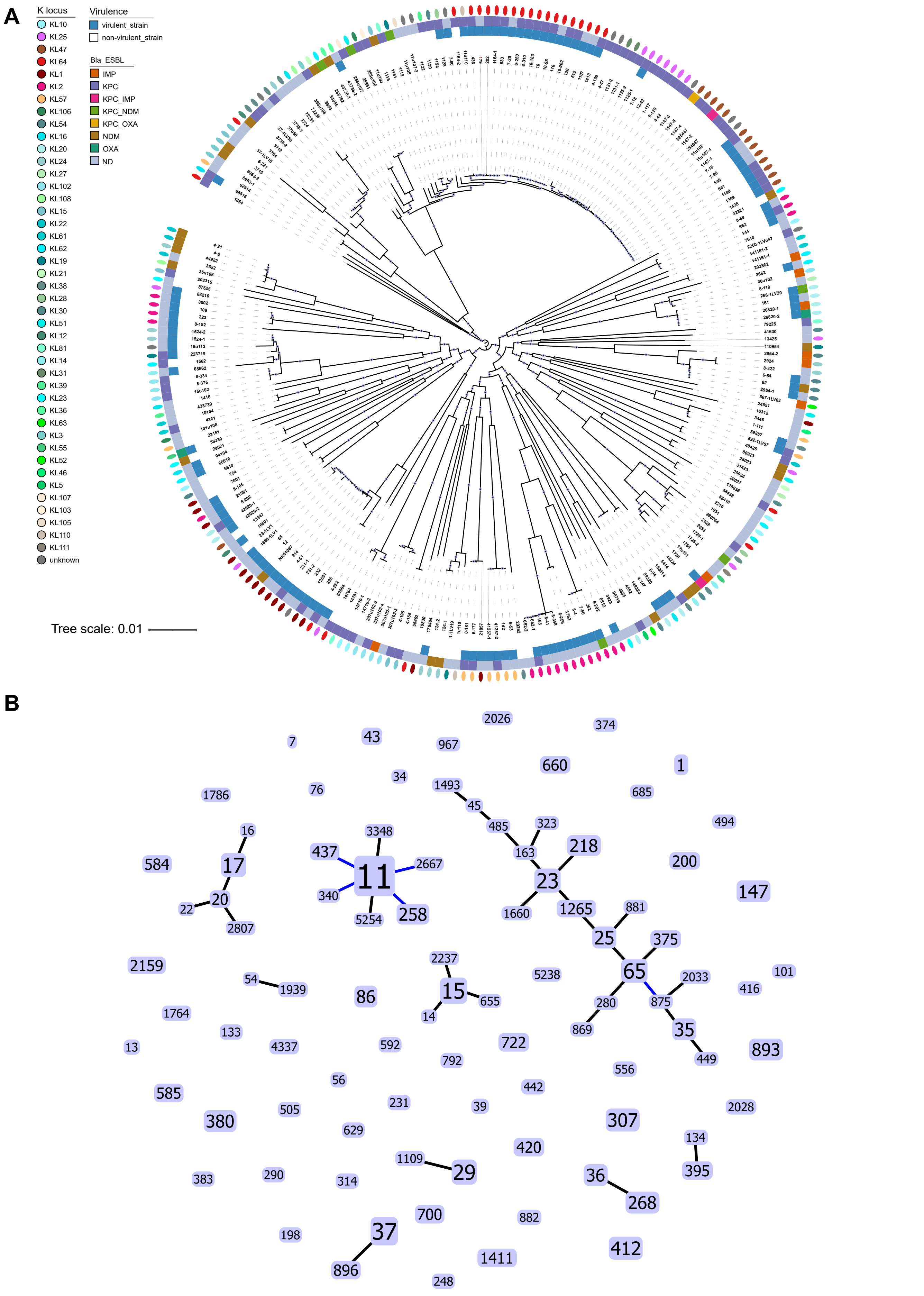


Figure S4. Phylogenetic tree and population snapshot of all 238 *K. pneumoniae* strains. A: Maximum-likelihood tree based on core genes of 238 *K. pneumoniae* strains clustered through midpoint rooting. Outer rings display virulent or non-virulent strains, extended-spectrum beta-lactamase genes, and KL types, respectively. B: Population snapshot generated using go-eBURST based on the MLST profiles of 238 *K. pneumoniae* strains. Each square represents an ST type. Relative sizes indicate the number of strains belonging to each ST. Numbers associated with the squares represent the assigned STs. Solid lines link single-locus variants that constitute a clonal complex.

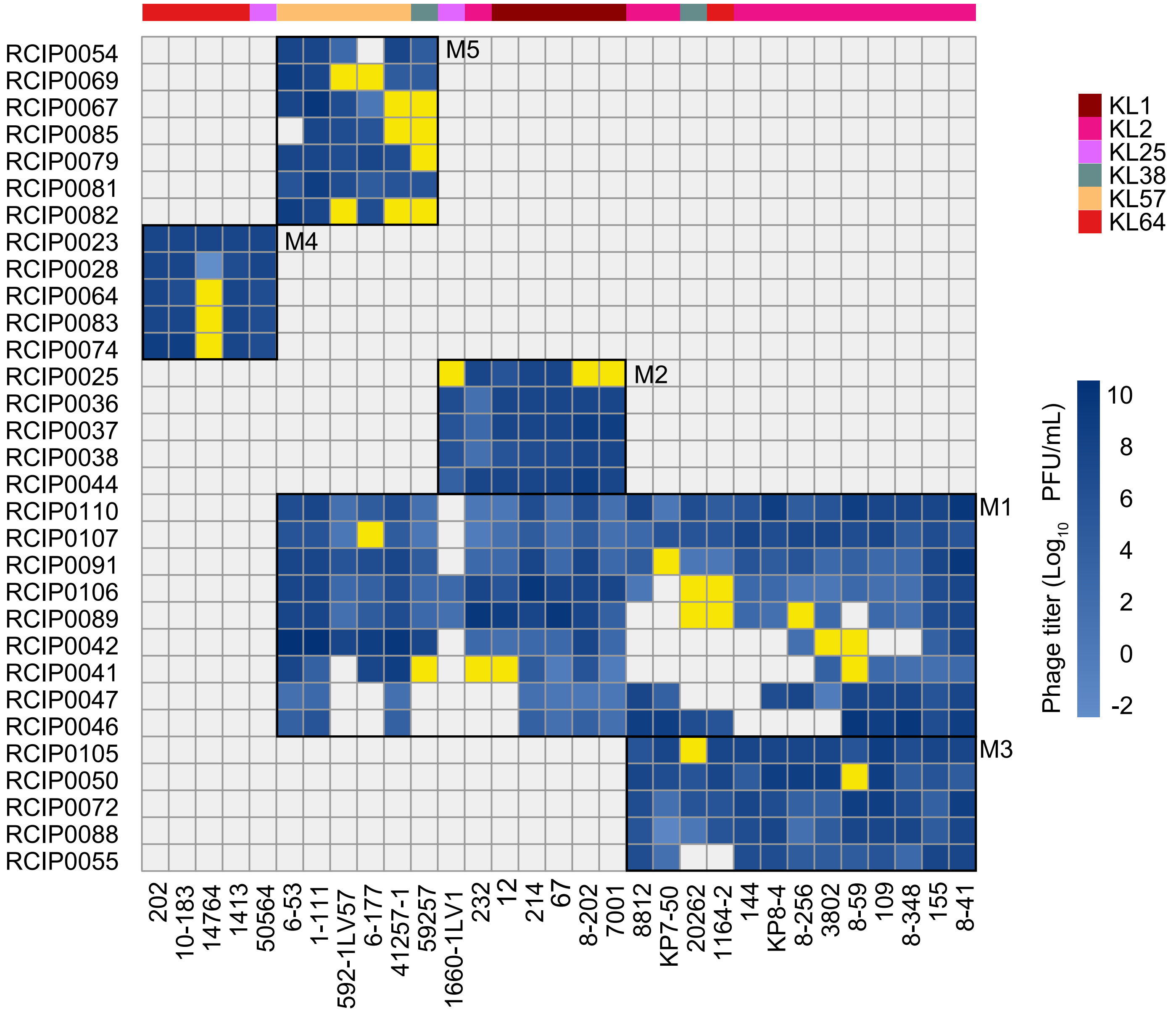


Figure S5. The phage-bacteria infection matrix, 353 lytic phenotypes from five distinct modules (M1, M2, M3, M4, and M5) in Figure 2A. The matrix is organized with bacterial hosts along the columns and phages along the rows, clustered by default parameters. Color legend denote successful phage-host infections, evidenced by double-layer plaque assay. Yellow squares denote successful phage-host lysis but are unable to generate offspring. Grey squares indicate that the phage is neither able to lyse nor produce offspring in the corresponding host. The initial titer of phages were normalized to $1\times10^8$[PFU]/mL.

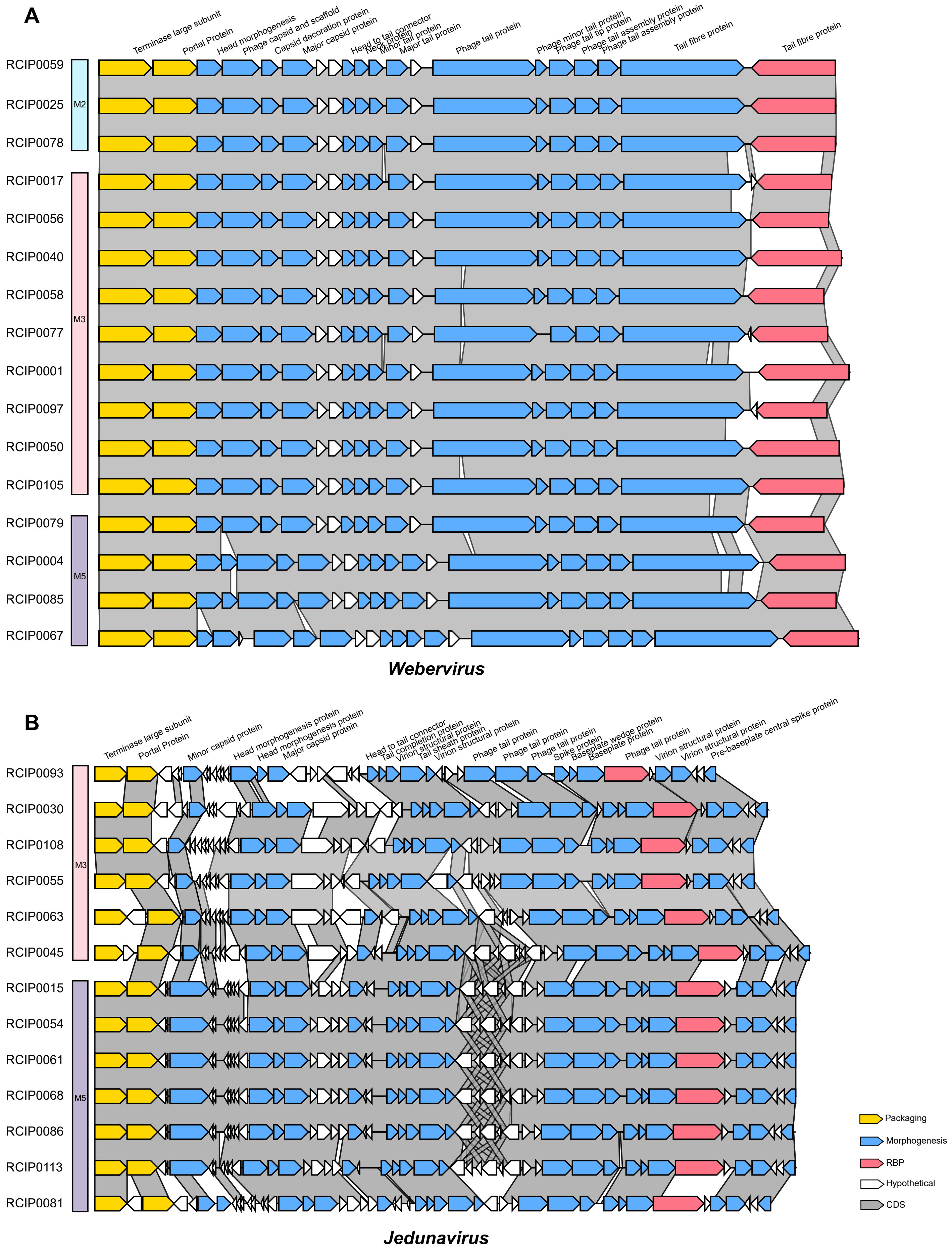
A
Terminase large subunit
Portal Protein
Head morphogenesis
Phage capsid and scaffold
Capsid decoration protein
Major capsid protein
Head to tail connector
Neck protein
Minor tail protein
Major tail protein
Phage tail protein
Phage minor tail protein
Phage tail tip protein
Phage tail assembly protein
Phage tail assembly protein
Tail fibre protein
Tail fibre protein
RCIP0059
RCIP0025
RCIP0078
RCIP0017
RCIP0056
RCIP0040
RCIP0058
RCIP0077
RCIP0001
RCIP0097
RCIP0050
RCIP0105
RCIP0079
RCIP0004
RCIP0085
RCIP0067
M2
M3
M5
Webervirus
B
Terminase large subunit
Portal Protein
Minor capsid protein
Head morphogenesis protein
Head morphogenesis protein
Major capsid protein
Head to tail connector
Tail completion protein
Virion structural protein
Tail sheath protein
Virion structural protein
Phage tail protein
Phage tail protein
Phage tail protein
Spike protein
Baseplate wedge protein
Baseplate protein
Phage tail protein
Virion structural protein
Virion structural protein
Pre-baseplate central spike protein
RCIP0093
RCIP0030
RCIP0108
RCIP0055
RCIP0063
RCIP0045
RCIP0015
RCIP0054
RCIP0061
RCIP0068
RCIP0086
RCIP0113
RCIP0081
M3
M5
Jedunavirus
Packaging
Morphogenesis
RBP
Hypothetical
CDS

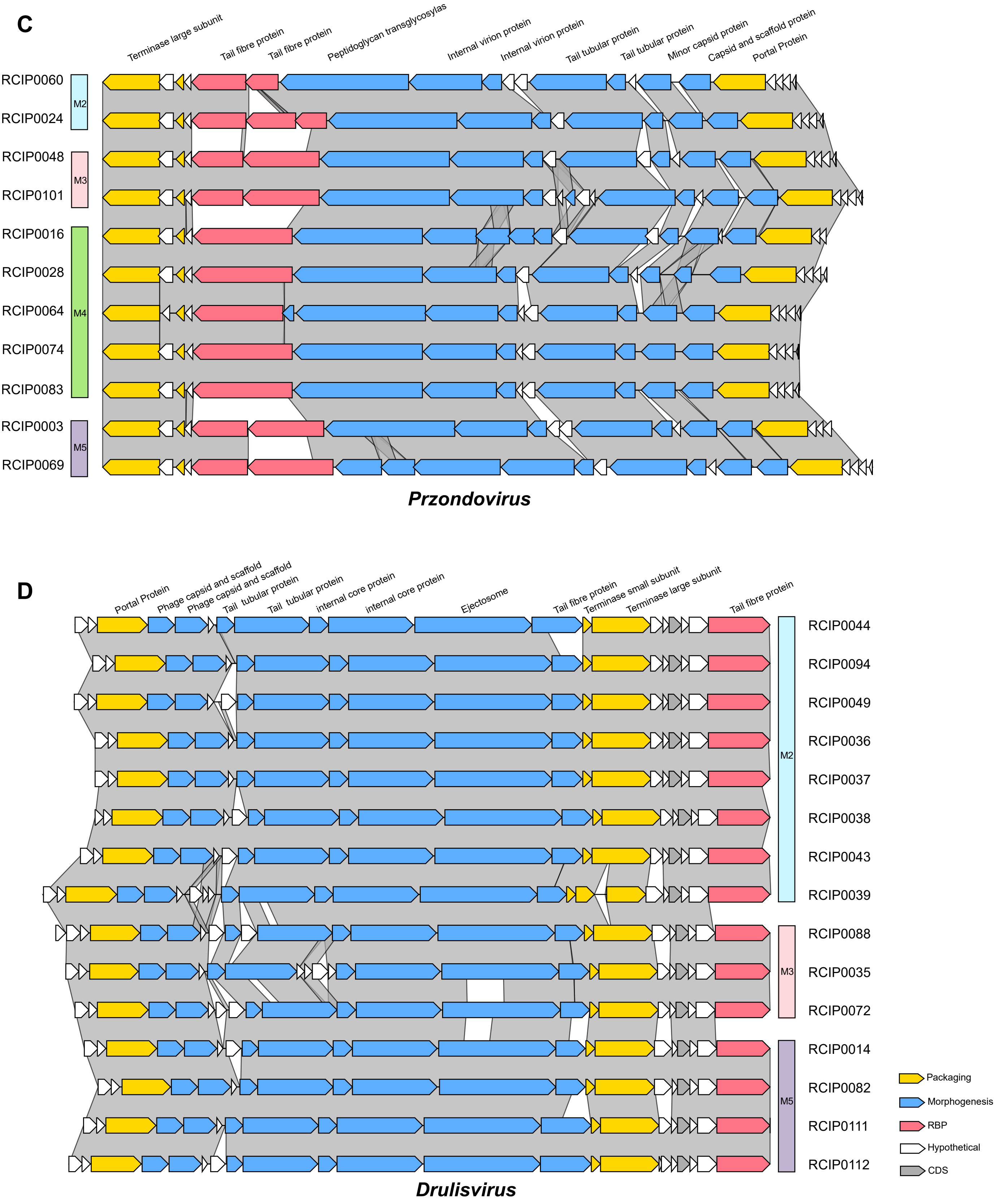


Figure S6. Comparative genomic analysis of the *Klebsiella phages* M2–M5, isolated from the same genus. ANI values were used to define similarity groups and to classify phages into the closest viral family, subfamily (if applicable), and genus via comparison with database sequences. The contigu-ous clusters encoding morphogenesis proteins from a given genus were aligned. A: *Webervirus*; B: *Jedunavirus*; C: *Przondovirus*; and D: *Drulisvirus*. The genome organisation of each phage is shown. Arrows represent coding sequences and are coloured according to the function of the encoded protein, as follows: yellow, packaging; blue, morphogenesis; deep pink, RBPs; grey, general func-tions; and white, hypothetical proteins. Grey shading represents >70% similarity at the nucleotide level.

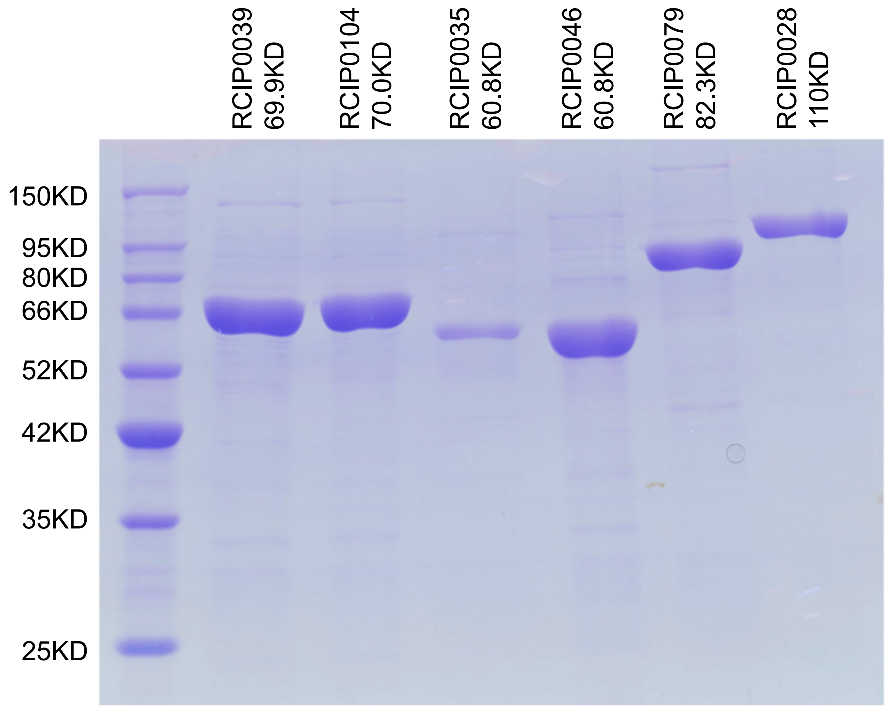


Figure S7. Sodium dodecyl sulphate polyacrylamide gel electrophoresis of purified RBPs. Lane 1, molecular weight markers (25–150 kDa); lane 2, purified RBP from RCIP0039; lane 3, purified RBP from RCIP0104; lane 4, purified RBP from RCIP0035; lane 5, purified RBP from RCIP0046; lane 6, purified RBP from RCIP0079; and lane 7, purified RBP from RCIP0028.

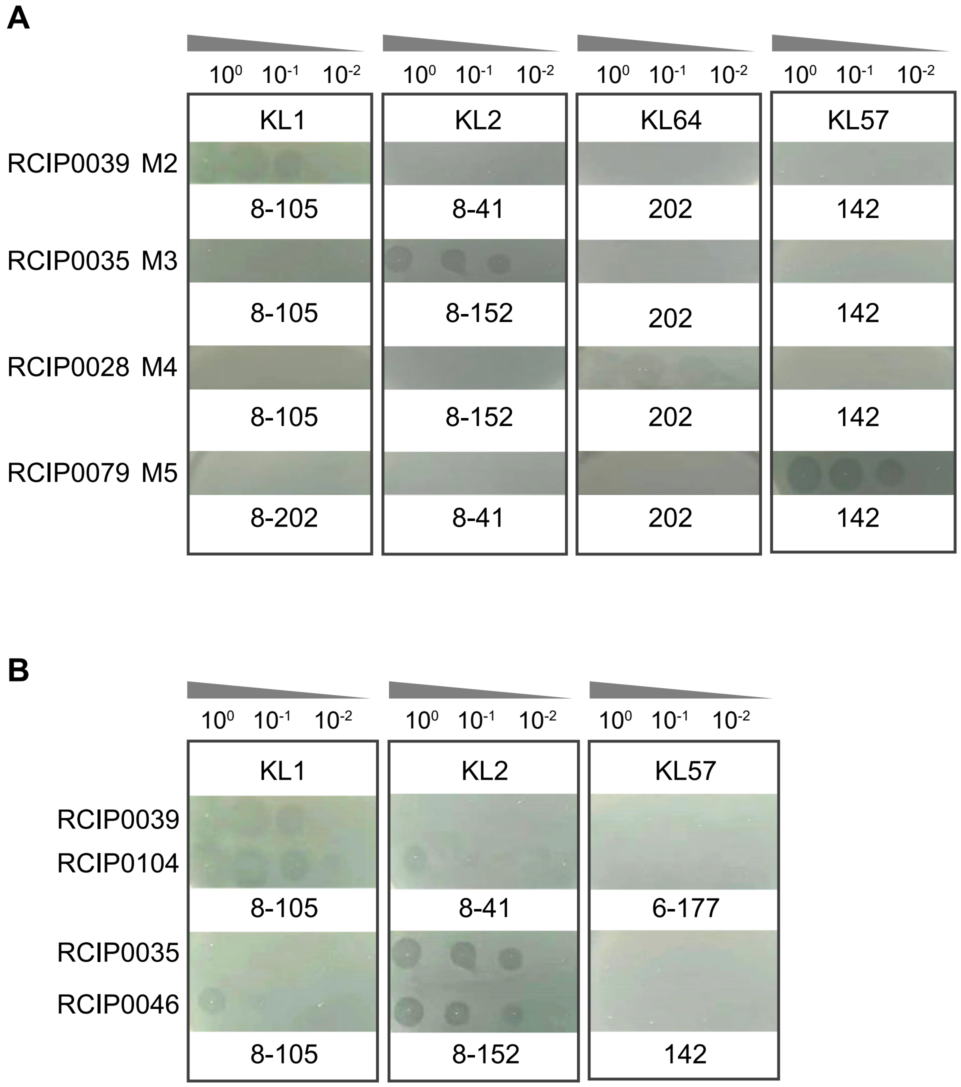


Figure S8. The activity of purified RBPs on *K. pneumoniae* strains. A: Spot tests were conducted using purified RBPs on lawns of *K. pneumoniae*. RBPI from phage RCIP0039, RBPIII from RCIP0035, RBPV from RCIP0028, and RBPVI from RCIP0079 were chosen for expression and purification to evaluate their activity. B: Spot tests were conducted using purified multivalent RBPs on lawns of *K. pneumoniae*. RBPI from phage RCIP0039, RBPIII from RCIP0035, RBPV from RCIP0028, and RBPVI from RCIP0079 were chosen for expression and purification to evaluate their activity.

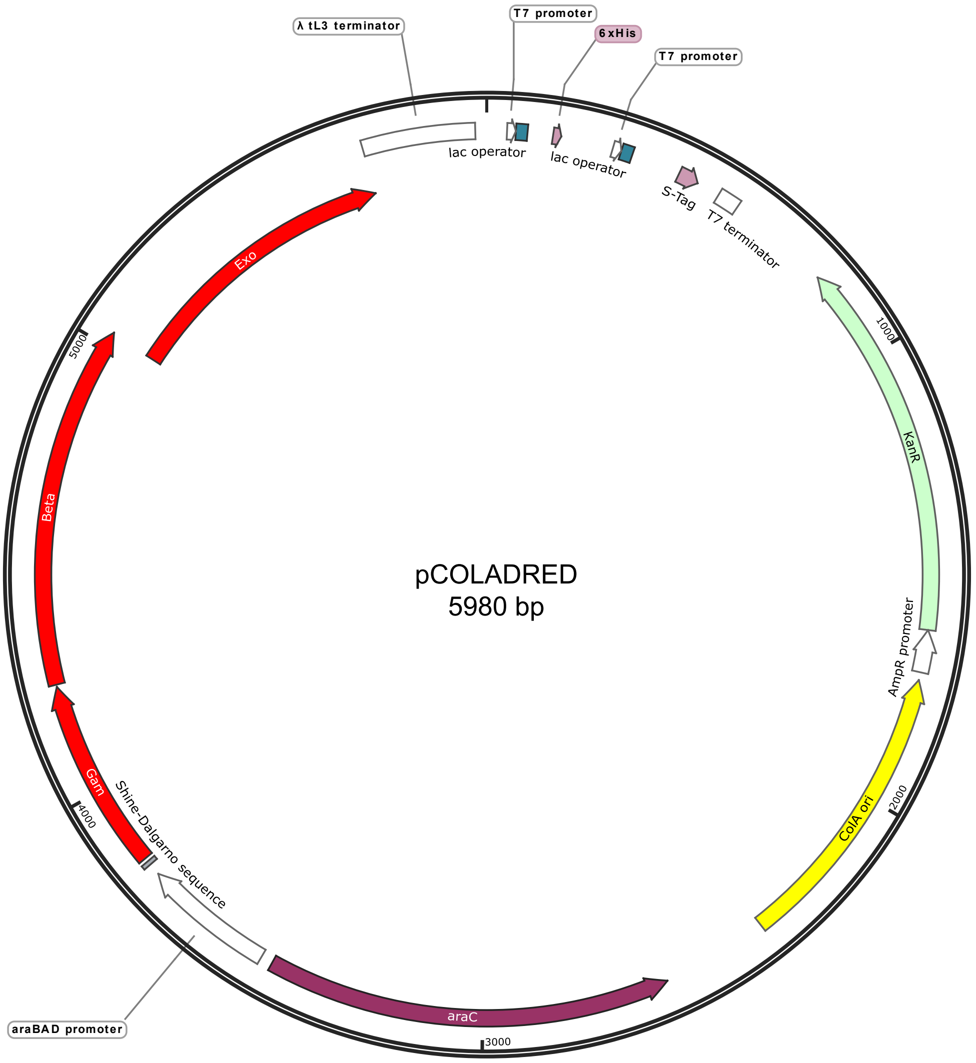


Figure S9. Plasmid structure and genetic context of pCOLADRed. Red arrows denote the lambda-derived Red recombination system.

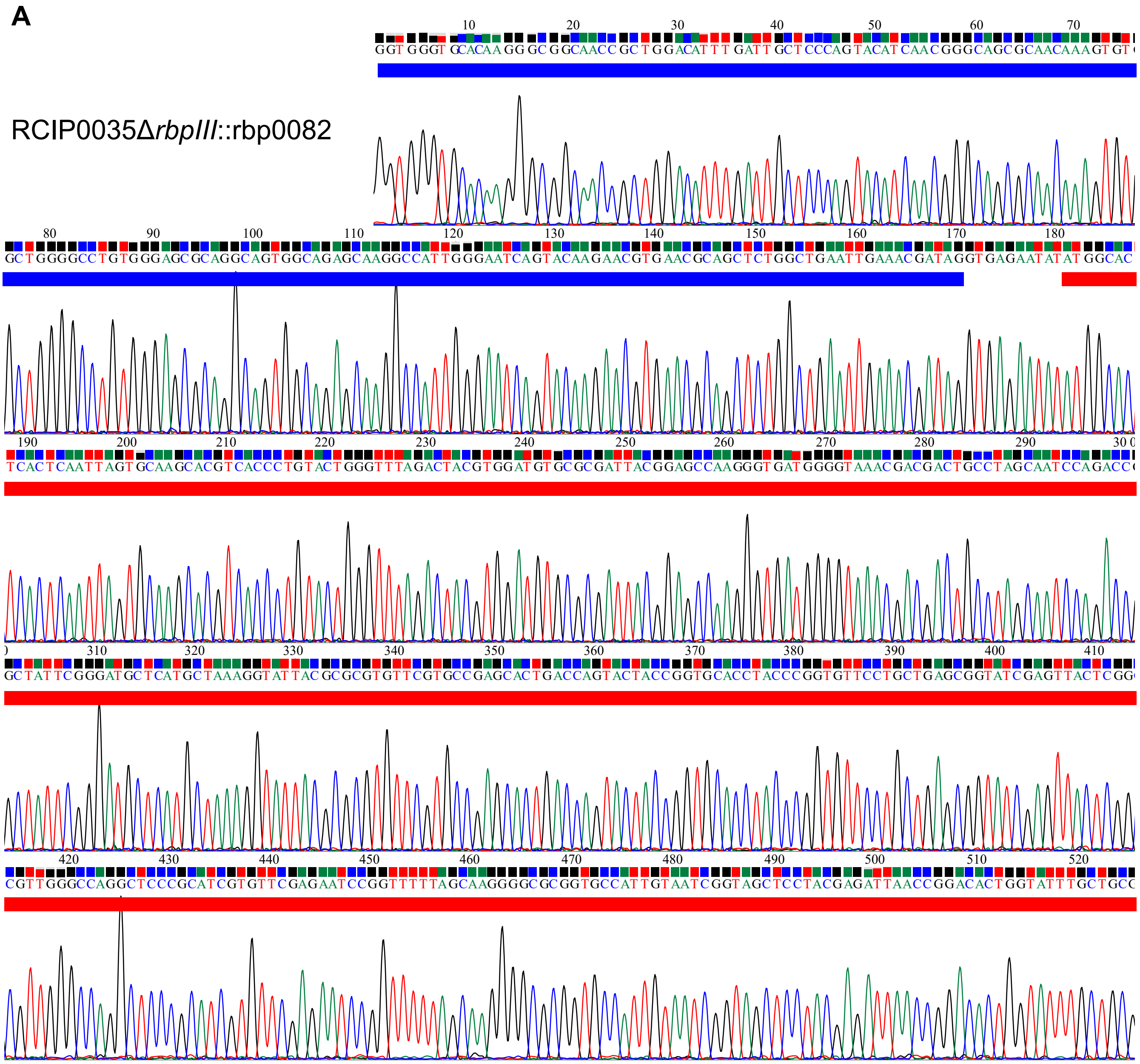
A
RCIP0035ΔrbpIII::rbp0082

**B**

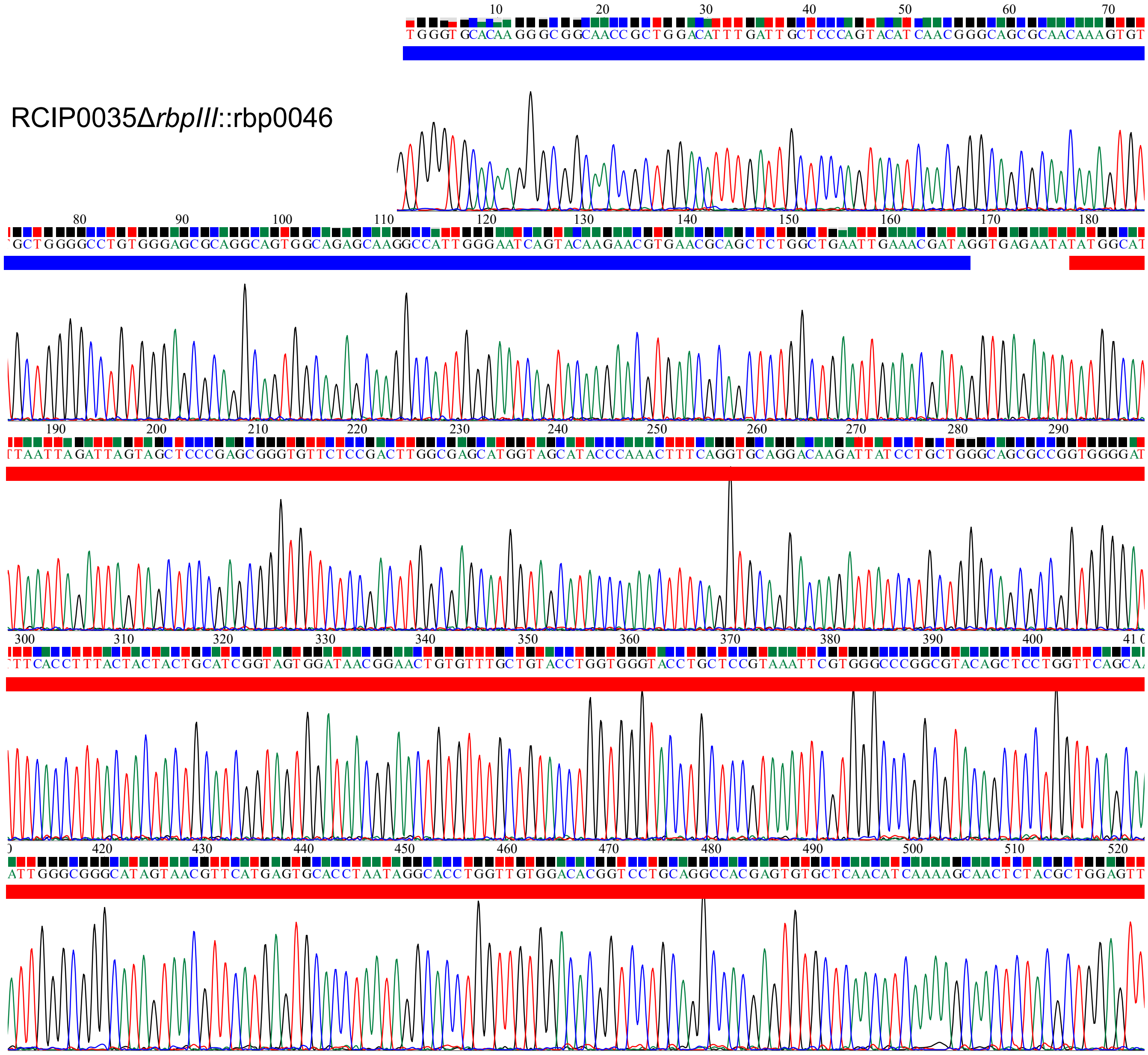

**C**

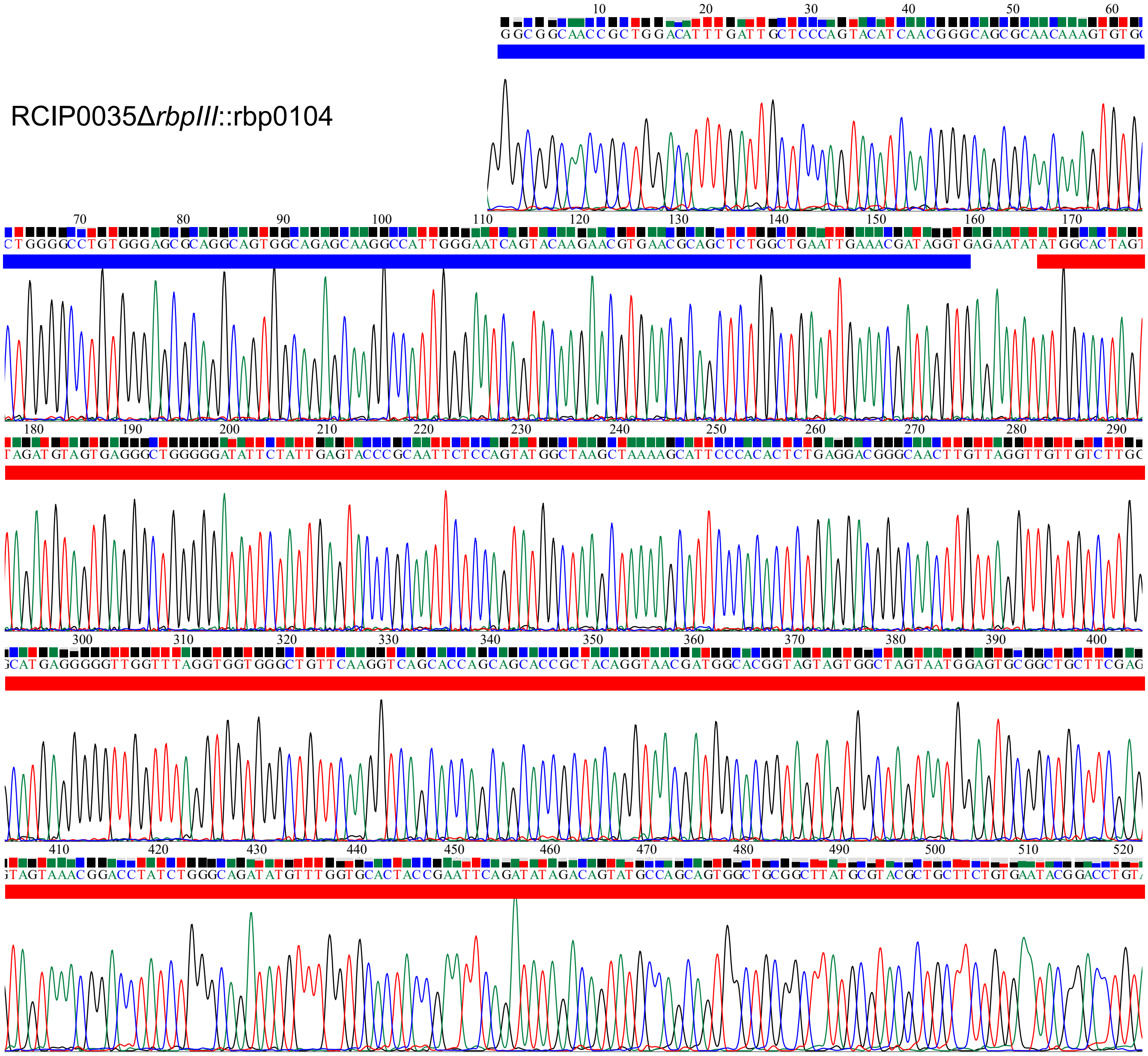


Figure S10. Sanger DNA sequencing of the recombinant phage. The blue band denotes the sequence of chasis phage RCIP0035 and the red band denotes the sequence of introduced RBP gene sequences. A: RCIP0035Δ*rbpIII*::rbp0082. B: RCIP0035Δ*rbpIII*::rbp0046. C: RCIP0035Δ *rbpIII*::rbp0104.

Table S1. Primers used to construct expression vectors.

| Primers | Primers' sequences |
|---|---|
| RCIP0035-F | CTGGTGCCGCGCGGCAGCCATATGGCACTAGTAGATTTAGTGAGGGC |
| RCIP0035-R | AGTGGTGGTGGTGGTGGTGCTCGAGTTACTTGACCACATTGCACAGC |
| RCIP0046-F | TGGTGCCGCGCGGCAGCCATATGGCACTAGTAGATGTAGTGAGGGC |
| RCIP0046-R | TGGTGGTGGTGGTGCTCGAGTTACTTGACCACGTTGCACAGCAAG |
| RCIP0039-F | TGGTGCCGCGCGGCAGCCATATGGCGTTAATTAGATTAGTAGCCCC |
| RCIP0039-R | TGGTGGTGGTGGTGCTCGAGTTAGAGATACACCTCCCAGGCTACC |
| RCIP0028-F | TGGTGCCGCGCGGCAGCCATATGGACCAAGATACTAAAACAATTATCCAGTAC |
| RCIP0028-R | TGGTGGTGGTGGTGCTCGAGTTATGCGTTCAGGTACACCCCG |
| RCIP0079-F | TGGTGCCGCGCGGCAGCCATATGGCACTATACAGAGAAGGCAAAGCG |
| RCIP0079-R | TGGTGGTGGTGGTGCTCGAGTTAGTCCCCCAAGTAGGTAAATTTGATCTCTTT |
| RCIP0104-F | TGGTGCCGCGCGGCAGCCATATGGCATTAATTAGATTAGTAGCTCCCG |
| RCIP0104-R | TGGTGGTGGTGGTGCTCGAGTTAGAGATACACCTCCCAGGCTACC |
| RCIP0046-F | TGGTGCCGCGCGGCAGCCATATGGCACTAGTAGATGTAGTGAGGGC |
| RCIP0046-R | TGGTGGTGGTGGTGCTCGAGTTACTTGACCACGTTGCACAGCAAG |

Table S2. Primers used for constructing recombinant phages with RBP replacement.

| Primers | Sequence (5'–3') |
|---|---|
| **RCIP0035-RBP→RCIP0082-RBP** | |
| RCIP0035-rbp-up-F | ATAAGAAGGAGATATACATATGAGACAGTGCCGGTATCCC |
| RCIP0035-rbp-up-R0082 | ACTAATTGAGTGAGTGCCATATATTCTCACCTATCGTTTCAATTC |
| RCIP0082-rbp-F | GAATTGAAACGATAGGTGAGAATATATGGCACTCACTCAATTAGTGCAAG |
| RCIP0082-rbp-R | ACAATGGCCAACAAGAGCCATTATTTGTACCCCGCTACTCGTAG |
| RCIP0035-rbp-down-F0082 | GAGTAGCGGGGTACAAATAATGGCTCTTGTTGGCCATTGTTGG |
| RCIP0035-rbp-down-R | TTTCTTTACCAGACTCGAGGGTACCAGTGGGGAGCGCGCAG |
| **RCIP0035-RBP→RCIP0046-RBP** | |
| RCIP0035-rbp-up-F | ATAAGAAGGAGATATACATATGAGACAGTGCCGGTATCCC |
| RCIP0035-rbp-up-R0046 | ACTACATCTACTAGTGCCATATATTCTCACCTATCGTTTCAATTC |
| RCIP0104-rbp-F | GAAACGATAGGTGAGAATATATGGCACTAGTAGATGTAGTGAGGG |
| RCIP0104-rbp-R | ACAATGGCCAACAAGAGCCATTACTTGACCACGTTGCACAGC |
| RCIP0035-rbp-down-F0046 | TGTGCAACGTGGTCAAGTAATGGCTCTTGTTGGCCATTGTTGG |
| RCIP0035-rbp-down-R | TTTCTTTACCAGACTCGAGGGTACCAGTGGGGAGCGCGCAGCG |
| **RCIP0035-RBP→RCIP0104-RBP** | |
| RCIP0035-rbp-up-F | ATAAGAAGGAGATATACATATGAGACAGTGCCGGTATCCC |
| RCIP0035-rbp-up-R0104 | ACTAATCTAATTAATGCCATATATTCTCACCTATCGTTTCAATTC |
| RCIP0104-rbp-F | GAAACGATAGGTGAGAATATATGGCATTAATTAGATTAGTAGCTC |
| RCIP0104-rbp-R | ACAATGGCCAACAAGAGCCATTAGAGATACACCTCCCAGGCTACC |
| RCIP0035-rbp-down-F0104 | CCTGGGAGGTGTATCTCTAATGGCTCTTGTTGGCCATTGTTGG |
| RCIP0035-rbp-down-R | TTTCTTTACCAGACTCGAGGGTACCAGTGGGGAGCGCGCAGCG |
| **RCIP0035 recombinants check Primers** | |
| Check Primers-F | CCAGCACAATGAGGGCTTGAGCC |
| Check Primers-R0082 | TACCGTATCCACCATCCGTA |
| Check Primers-R0046 | CAGGGCTGGGCTAAGGTTGT |
| Check Primers-R0104 | GCCCACCGTCACACAGCTTG |

**Table S3. Bacteria and plasmids used in this study.**

| Bacterial strains and plasmids | Relevant characteristics | Source |
|---|---|---|
| **Bacterial strains** | | |
| *E. coli* TOP10 | $F^-$ *mcr* A Δ(*mrr*-*hsd* RMS-*mcr* BC) φ80 *lac* Z ΔM15 Δ*lac* X74 *rec* A1 *ara* Δ139 Δ (*ara*-leu)7697 *gal* U *gal* K *rps* L($str^r$) *end* A1 *nup* G | ZOMANBIO(Beijing, China) |
| *E. coli* BL21(DE3) | $F^-$ *omp* T *hsd* S($rB^-mB^-$) *gal dcm* (DE3) | ZOMANBIO(Beijing, China) |
| **Plasmids** | | |
| pET-28a(+) | $Km^R$, T7/*lac* -promoter | Novagen (Madison, USA) |
| pCOLADuet-1 | $Km^R$, T7/*lac* -promoter | Novagen (Madison, USA) |
| pET28a::*rbp* III-RCIP0035 | $Km^R$, T7-promoter, expressing RBPIII-RCIP0035 | This study |
| pET28a::*rbp* I-RCIP0039 | $Km^R$, T7-promoter, expressing RBPI-RCIP0039 | This study |
| pET28a::*rbp* I-RCIP0104 | $Km^R$, T7-promoter, expressing RBPI-RCIP0104 | This study |
| pET28a::*rbp* III-RCIP0046 | $Km^R$, T7-promoter, expressing RBPIII-RCIP0046 | This study |
| pET28a::*rbp* VI-RCIP0079 | $Km^R$, T7-promoter, expressing RBPVI-RCIP0079 | This study |
| pET28a::*rbp* VI-RCIP0028 | $Km^R$, T7-promoter, expressing RBPVI-RCIP0028 | This study |
| pCOLADRed | $Km^R$, *ara* BAD promoter, expressing lambda-derived Red recombination system | This study |
| pCOLADRedRE0082 | $Km^R$, *ara* BAD promoter, expressing lambda-derived Red recombination system, carrying the upstream and downstream regions surrounding the RBPIII-RCIP0035 gene, and the RBPVI-RCIP0082 gene | This study |
| pCOLADRE0046 | $Km^R$, *ara* BAD promoter, expressing lambda-derived Red recombination system, carrying the upstream and downstream regions surrounding the RBPIII-RCIP0035 gene, and the RBPIII-RCIP0046 gene | This study |
| pCOLADRE0104 | $Km^R$, *ara* BAD promoter, expressing lambda-derived Red recombination system, carrying the upstream and downstream regions surrounding the RBPIII-RCIP0035 gene, and the RBPI-RCIP0104 gene | This study |
